# Involvement of RIG-I Pathway in Neurotropic Virus-Induced Acute Flaccid Paralysis and Subsequent Spinal Motor Neuron Death

**DOI:** 10.1128/mBio.02712-21

**Published:** 2021-11-16

**Authors:** Meenakshi Bhaskar, Sriparna Mukherjee, Anirban Basu

**Affiliations:** a National Brain Research Centre, Manesar, Haryana, India; CDC

**Keywords:** acute flaccid paralysis, neurotropic virus, motor neuron, cell death, pattern-recognition receptor RIG-I

## Abstract

Poliomyelitis-like illness is a common clinical manifestation of neurotropic viral infections. Functional loss and death of motor neurons often lead to reduced muscle tone and paralysis, causing persistent motor sequelae among disease survivors. Despite several reports demonstrating the molecular basis of encephalopathy, the pathogenesis behind virus-induced flaccid paralysis remained largely unknown. The present study for the first time aims to elucidate the mechanism responsible for limb paralysis by studying clinical isolates of Japanese encephalitis virus (JEV) and Chandipura virus (CHPV) responsible for causing acute flaccid paralysis (AFP) in vast regions of Southeast Asia and the Indian subcontinent. An experimental model for studying virus-induced AFP was generated by intraperitoneal injection of 10-day-old BALB/c mice. Progressive decline in motor performance of infected animals was observed, with paralysis being correlated with death of motor neurons (MNs). Furthermore, we demonstrated that upon infection, MNs undergo an extrinsic apoptotic pathway in a RIG-I-dependent fashion via transcription factors pIRF-3 and pIRF-7. Both gene-silencing experiments using specific RIG-I-short interfering RNA and *in vivo* morpholino abrogated cellular apoptosis, validating the important role of pattern recognition receptor (PRR) RIG-I in MN death. Hence, from our experimental observations, we hypothesize that host innate response plays a significant role in deterioration of motor functioning upon neurotropic virus infections.

## INTRODUCTION

Virus-induced acute flaccid paralysis (AFP) or viral myelitis, including complex poliomyelitis-like syndrome, a term coined by T. Solomon in 1998 (1), has emerged as a serious health concern after being rereported in various forms of epidemics annually (2–10). Despite the success of the World Health Organization’s global polio eradication initiative, AFP remains a prevalent disease in children below the age of 15 (11, 12). Active surveillance of AFP following the polio vaccine era has linked the unusual rise of poliomyelitis cases to non-polio viral infections (13–15). Asymmetric flaccid weakness, a clinical manifestation of AFP, may progress to permanent limb paralysis in severe-outcome patients, adding to disability-adjusted life years (16). Owing to its clinical importance with no specific treatment available, a lack of an experimental model to understand the etiology of virus-induced AFP further limits the development of potential therapies.

Like polio, many emerging viral infections, such as enterovirus, West Nile virus (WNV), and Japanese encephalitis virus (JEV), are causing AFP outbreaks annually due to endemic circulation of virus among populations (17–22). JEV and CHPV are two such viruses, endemic to Southeast Asia and the Indian subcontinent, which are mainly responsible for causing AFP and encephalitis in young children (23). Patients suffering from JEV and CHPV principally develop flu-like symptoms and characteristic features of lower motor neuron (MN) disease, such as lower limb wastage and flaccid weakness in affected populations (24, 25). Historically, the first clinical case of JEV-induced AFP was identified in 1945 on Okinawa Island, where the native population presented features of flaccid paralysis with sudden onset of fever and headache. When observed microscopically, autopsy samples of JE-positive patients displayed damaged anterior horn cells that were histologically similar to the lesions observed in acute poliomyelitis disease (26). Extensive electrophysiological and radiological studies performed on conscious JE-infected patients further suggested a role of anterior horn cells in JEV-induced muscle wasting (27). Additionally, magnetic resonance imaging (MRI) scans showed abnormal signal intensity on T2-weighted images of spine, indicating anterior horn cell injury in convalescent patients (28). Consistent with the clinical case reports, the anatomical basis of virus-induced AFP was first demonstrated in mice after neuroadapted Sindbis virus infection (29). Later, various studies conducted showed evidence of apoptotic injury in mouse central nervous system (CNS) upon EV71, WNV, reovirus, and Sindbis virus infection (30–34); however, the precise mechanism behind MN death and virus-induced AFP remained elusive.

Research in our laboratory previously defined a role of oxidative stress and neuroinflammation in brain, which answered some of the basic questions of virus-induced encephalitis (35–40). Therefore, in this study, we developed an experimental model of virus-induced AFP that replicates all features of clinical AFP. Interestingly, we found that viral replication in lumbar cord correlates with behavioral impairments and paralysis in diseased mice, with RIG-I signaling playing a central role in activating the extrinsic apoptotic pathway. Thus, overall, we addressed two important questions of viral AFP. First, we described that sustained activation of RIG-I/pIRF3-7 signaling upon infection triggers the host endogenous apoptotic pathway, which, being a part of the antiviral response system, leads to MN death. Second, our investigations further highlighted that RIG-I-induced MN death during neurotropic virus infection is independent and distinct from well-described type 1 interferon (IFN)-induced apoptosis.

## RESULTS

### Characterization of neurotropic virus-induced AFP.

**FIG 1:**
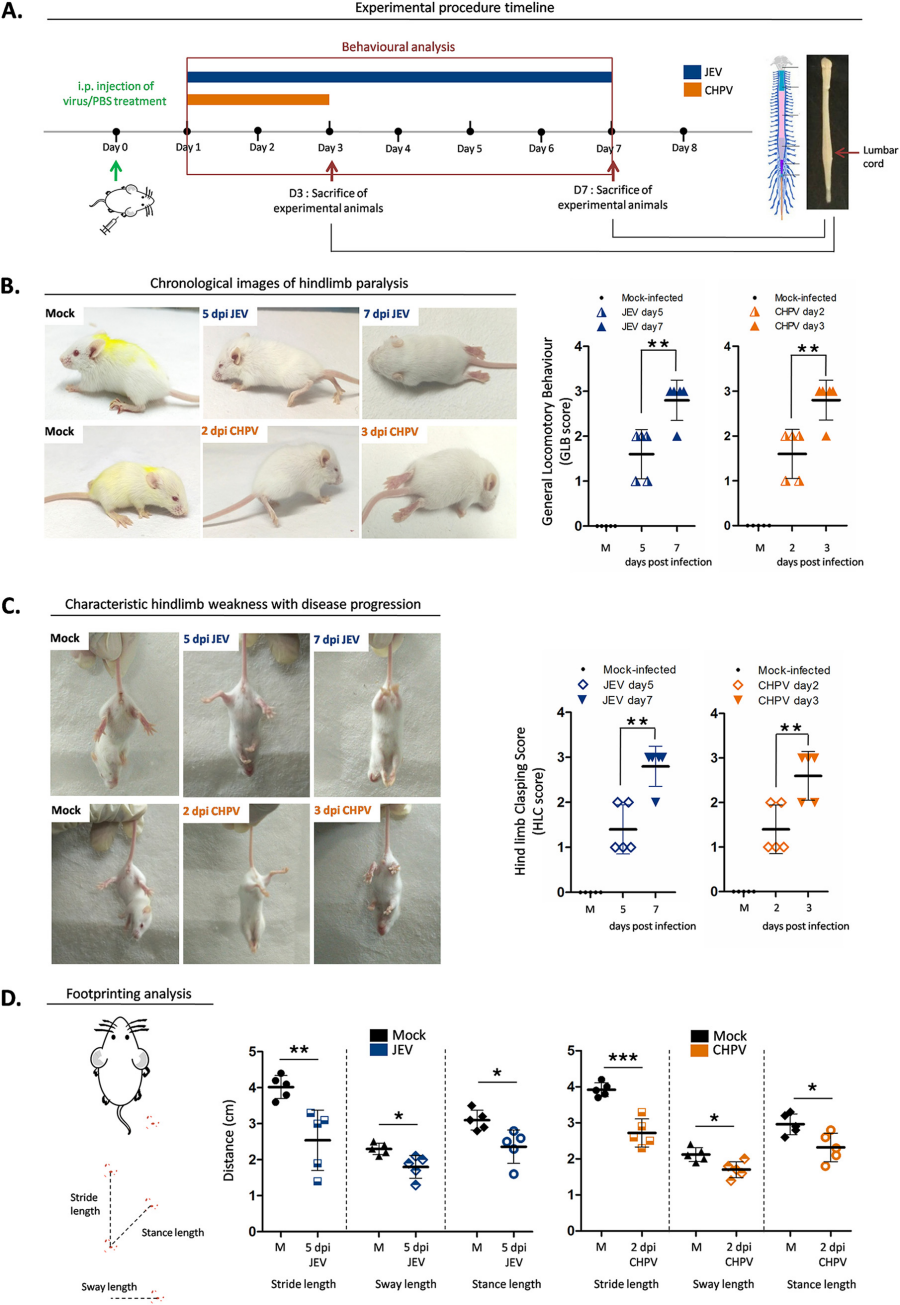
Japanese encephalitis virus and Chandipura virus induce AFP in 10-day-old BALB/c mice. (A) Pups were either mock infected using PBS or inoculated intraperitoneally with 3 × 10^4^ PFU of JEV or 1 × 10^4^ PFU of CHPV. Animals were scored daily until clinical features of hindlimb paralysis appeared. Pups were sacrificed and lumbar cord tissue was harvested at days 1, 3, 5, and 7 postinfection (pi) for JEV and days 1, 2, and 3 pi for CHPV. Blue bar charts represent JEV infection and orange bar charts represent CHPV infection. (B) Characteristic changes in locomotion of infected animals with representative posture of duck foot motion and hindlimb paralysis were scored quantitatively and analyzed as GLB (general locomotory behavior) score. Data are represented as means ± standard deviations (SD) for 5 animals per group where *P* values were determined (*, *P* < 0.05; **, *P* < 0.01; ***, *P* < 0.001) using one-way ANOVA followed by *post hoc* Bonferroni test. (C) Altered hindlimb clasping phenotype with typical posture of hindlimbs retracted toward the midline or away from midline was scored as HLC (hindlimb clasping) score where data are represented as means ± SD for 5 animals per group, with statistical significance being determined (*, *P* < 0.05; **, *P* < 0.01; ***, *P* < 0.001) using one-way ANOVA followed by *post hoc* Bonferroni correction. (D) Severity in gait of the animals was quantified by footprinting analysis where error bars are represented as mean ± SD for 5 animals, with *P* values determined (*, *P* < 0.05; **, *P* < 0.01; ***, *P* < 0.001) using two-tailed paired *t* test.

In this study, we screened two clinical isolates of neurotropic virus, JEV (strain GP-78) and CHPV (strain 1653514), for their ability to induce flaccid paralysis in BALB/c mice (Fig. 1A). Both viral strains induced AFP by day 5 postinfection (pi) for JEV and day 2 pi for CHPV in mice. The degree of virus-induced paralysis was accurately determined by performing series of behavioral tests. Animals were scored on a scale of 0 to 3 to study general locomotion in mice (GLB score) (Fig. 1B), ranging from the ability to walk freely to no movement at all. Postinfection, both experimental groups showed significant differences in GLB score compared to respective age-matched controls. For JEV, 3/5 animals studied displayed hind leg inhibition with severe limp at day 5 of infection, while 2/5 animals exhibited features of tiptoe or knuckle walking. With disease progression (7 days pi [dpi]), 4/5 animals in the JEV group exhibited complete paralysis of hindlimbs (GLB score = 3) with zero movement, while 1/5 displayed severe limping. Akin to the JEV group, mice infected with CHPV also showed significantly higher GLB scores than mock-infected animals. For CHPV, 3/5 mice developed hind leg inhibition with severe limp shortly after infection (2 dpi), while 4/5 mice showed complete paralysis of lower limbs by day 3 pi. To further assess MN degeneration, hindlimb clasping score (HLC) (Fig. 1C) was calculated, wherein the infected group again displayed significantly higher scores than their control littermates. Interestingly, from both the JEV and CHPV experimental groups, 2/5 animals developed partial retraction of hindlimbs toward midline when suspended freely in air, while with progression of infection, 4/5 animals in the JEV and 3/5 animals in the CHPV group developed complete retraction of hindlimbs (HLC score, 3). In a further attempt to analyze gait abnormalities, we did footprint analysis, wherein significant decrease in stride length, sway length, and stance length were recorded compared with mock-infected animals (Fig. 1D).

**FIG 2:**
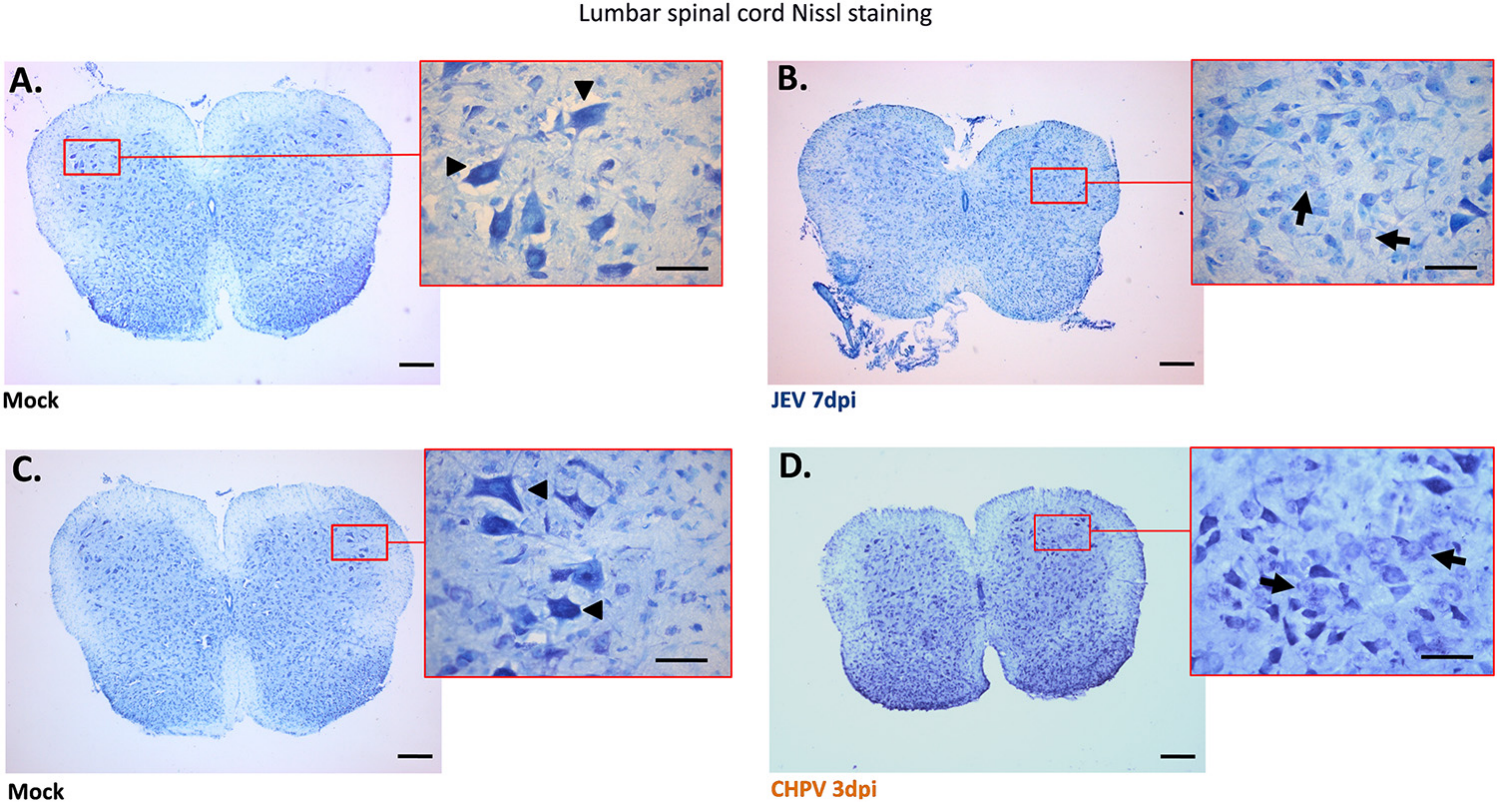
Pathology of lumbar motor neurons (MNs) following JEV and CHPV infection in mice. Ten-day-old BALB/c pups were either mock infected with PBS or inoculated intraperitoneally with 3 × 10^4^ PFU of JEV or 1 × 10^4^ PFU of CHPV in a 25-μl volume. Animals were examined daily and sacrificed at day 7 pi for JEV and day 3 pi for CHPV, when pups displayed complete hindlimb paralysis with difficult or no movement at all. Thin sections of 20-μm thickness prepared from both mock-infected and virus-infected lumbar cord tissue were examined using Nissl stain. (A and C) Coronal lumbar cord sections of respective age-matched control animal (mock infected) with red-outlined boxes representing enlarged images of anterior horn SC (spinal cord) with healthy MNs indicated by black arrowheads. (B and D) Representative image of infected lumbar cord sections upon JEV and CHPV infection with magnified area displaying dying MNs indicated by black arrows. Scale bar denotes 50 μm and 200 μm using oil magnification. Data are representative of a minimum of three independent experiments performed.

To further correlate clinical observations of AFP with motor neuron pathology, we performed Nissl staining using spinal cord (SC) sections. Selective loss of motor neurons was observed exclusively in anterior horn of the lumbar region compared with cervical and thoracic SC sections of the same animal (data not shown). Both JEV- and CHPV-infected lumbar sections displayed features of chromatolysis, like eccentric nucleus and cellular swelling of MN (Fig. 2B and D), compared to healthy MNs observed in mock-infected mice (Fig. 2A and C). Thus, collectively these findings suggest that virus infection not only impaired overall motor coordination but also induced clinical AFP with degenerating motor neurons.

### Virus titer in lumbar SC corresponds to disease progression.

**FIG 3:**
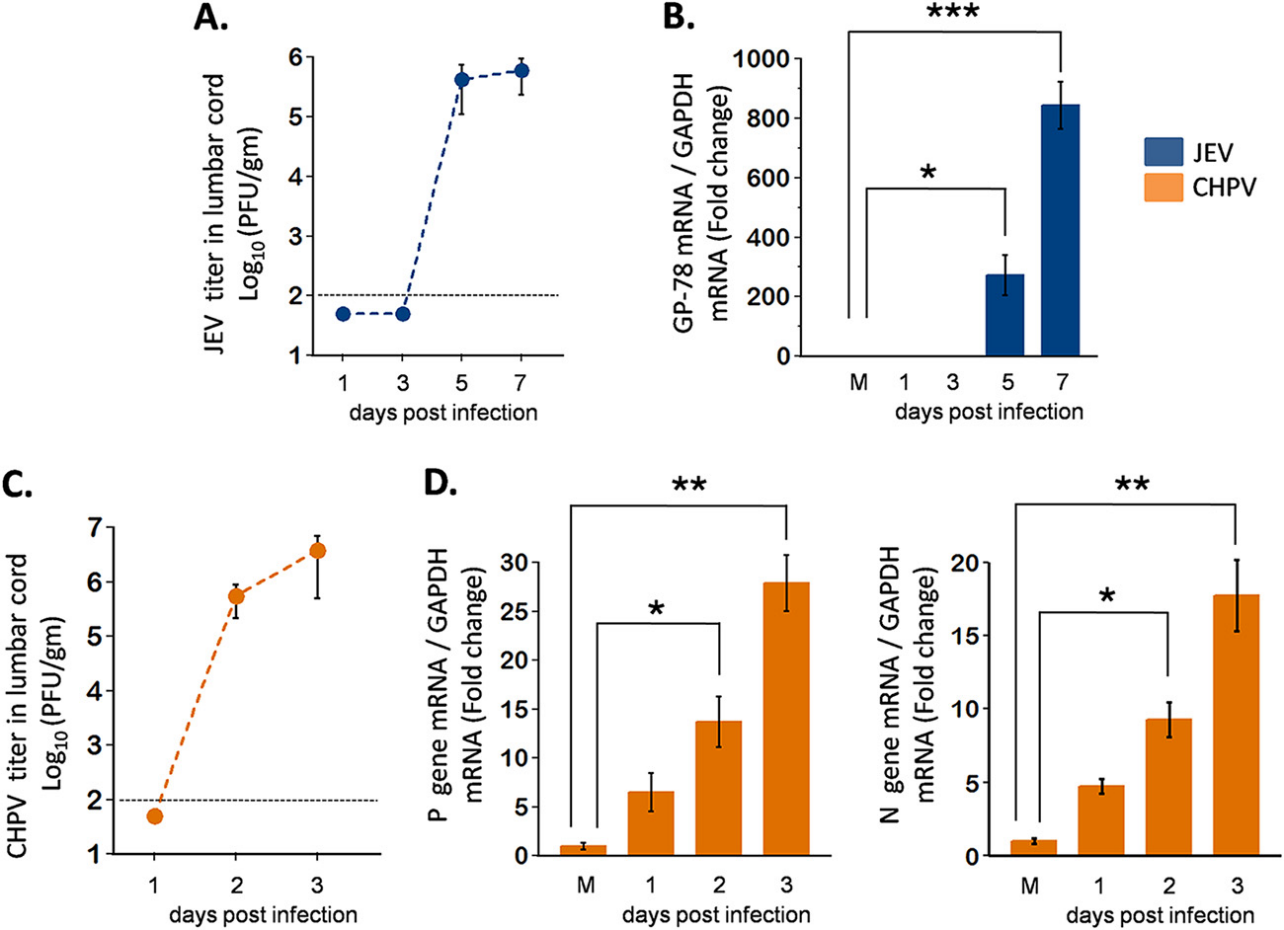
Viral titer in lumbar spinal cord (SC) of infected mice. Ten-day-old BALB/c pups were either PBS injected or infected with clinical isolates, 3 × 10^4^ PFU of JEV or 1 × 10^4^ PFU of CHPV, intraperitoneally. Viral titers were determined from infected lumbar cord tissue in a daywise manner (days 1, 3, 5, and 7 after JEV infection and days 1, 2, and 3 after CHPV infection) for studying viral kinetics with disease progression. Blue bar charts represent JEV-infected data and orange bar charts represent CHPV-infected data. (A and C) Lumbar tissue homogenates prepared and pooled from 2 mice for each time point of infection were analyzed for infectious virus count using plaque assay. Dashed lines represent the limit of detection of plaque assays for all experiments performed. (B and D) Total RNA was extracted from lumbar SC tissue for analyzing relative abundance of viral mRNA using qRT-PCR, where GAPDH was used as the loading control. Data represent mean ± standard deviation (SD) from a minimum of 3 independent experiments, where *P* values were determined (*, *P* < 0.05; **, *P* < 0.01; ***, *P* < 0.001) using one-way ANOVA followed by *post hoc* Bonferroni test.

To determine whether active replication of virus is associated with disease severity, we next performed plaque assays using lumbar cord homogenates at various dpi. To our interest, infectious viral particles were detected only at day 5 and 7 pi for JEV and day 2 and 3 pi for CHPV, suggesting inability of virus to penetrate the blood-brain barrier (BBB) at early dpi (Fig. 3A and C). Additionally, to confirm *in vivo* viral replication, two-step quantitative PCRs targeting glycoprotein gene of JEV and P, N gene of CHPV were performed to detect viral RNA extracted from lumbar cords at various time points pi. Significant upregulation of GP-78 (Fig. 3B) at days 5 and 7 pi for JEV and P, N gene (Fig. 3D) at days 2 and 3 pi for CHPV were detected. Overall, results from the quantitative real-time PCR (qRT-PCR) analysis and plaque assay paralleled the behavioral deficits observed in mice, indicating an association between viral replication and paralysis onset.

### JEV and CHPV infect motor neuron both *in vivo* and *in vitro*.

**FIG 4:**
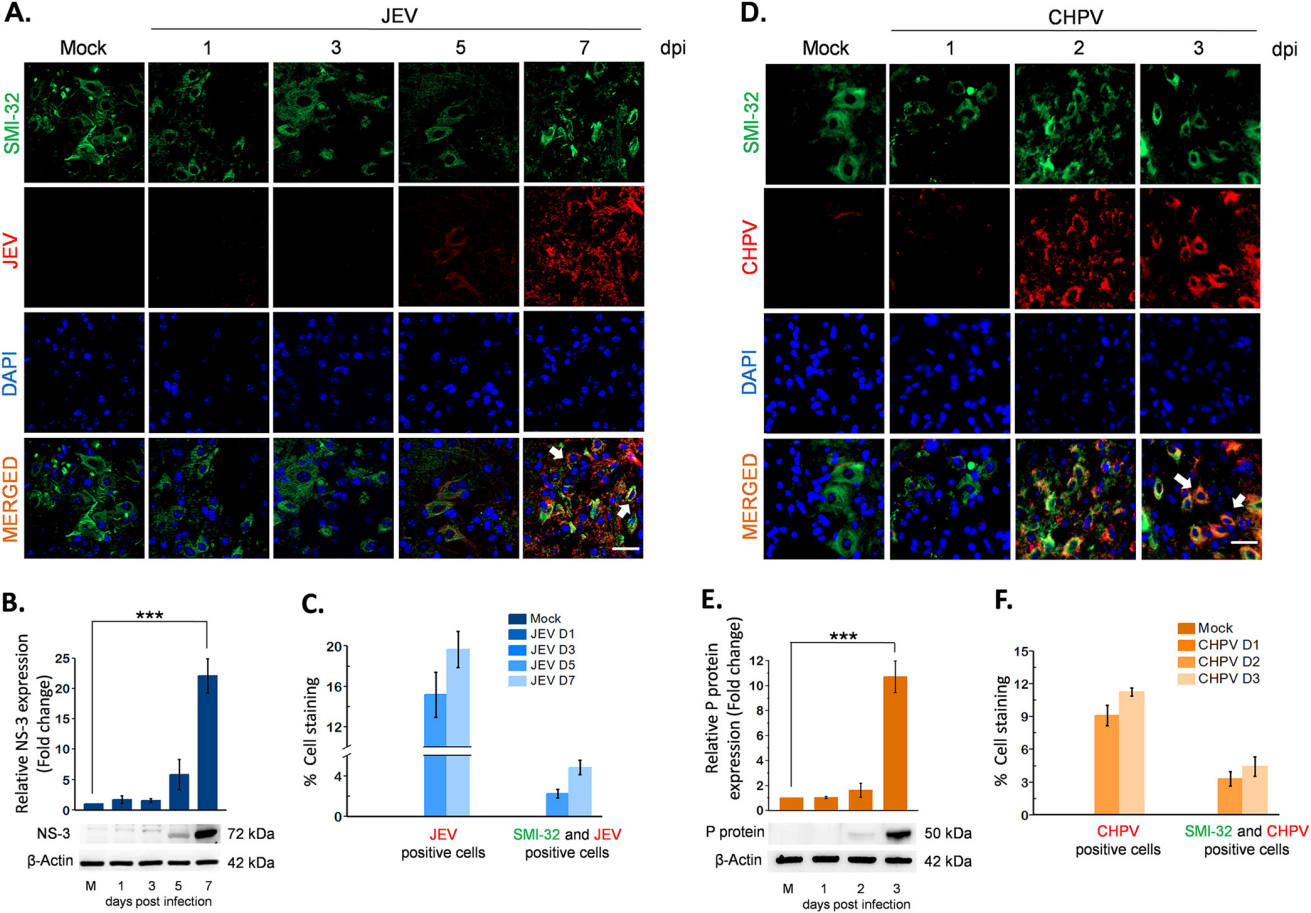
Virus-induced AFP correlates with spread of viral antigen in lumbar motor neurons. Ten-day-old BALB/c mice were either mock infected or inoculated intraperitoneally with 3 × 10^4^ PFU of JEV or 1 × 10^4^ PFU of CHPV. Infected cord tissues were collected in a daywise manner as indicated for studying paralysis onset and disease progression. Blue bar charts represent JEV infected data, and orange bar charts represent CHPV infected data. (A and D) Lumbar cord sections were double stained for motor neuron marker SMI-32 and viral antigen NS3 for JEV and P-protein for CHPV infection. Representative epifluorescence images show colocalization of viral antigen with MN marker and DAPI, indicated by white arrows in respective panels. Scale bar denotes 50 μm. (B and E) Lumbar cord lysates from both PBS-injected and virus-infected pups were analyzed using Western blotting to determine relative abundance of viral proteins (NS3 for JEV and P-protein for CHPV). (C and F) Representative graphs showing percentage of JEV- and CHPV-infected cells along with double-positive cells for SMI32 (motor neuron marker) and viral protein markers in respective lumbar cord sections. Data here represent mean ± SD from a minimum of 3 independent experiments, where statistical significance (*, *P* < 0.05; **, *P* < 0.01; ***, *P* < 0.001; NS, nonsignificant) was calculated using one-way ANOVA followed by *post hoc* Bonferroni correction.

To determine whether virus-induced AFP is linked to active infection of MNs, we performed immunohistochemistry (IHC) with lumbar sections using anti-JEV NS3 and anti-CHPV P-protein antibody. Motor neurons were identified using anti-SMI32, a specific marker for anterior horn cells, which appeared intact with no immunoreactivity toward viral antigen in mock-infected samples. Both JEV- and CHPV-infected pups displayed clear colocalization of SMI32 and NS3 and P-protein at days 5 and 7 pi for JEV and days 2 and 3 pi for CHPV (Fig. 4A and D), with quantification graphs attached (Fig. 4C and F). Interestingly, viral protein kinetics (blots) showed significant upregulation of NS3 at day 7 pi for JEV and P-protein at day 3 pi for CHPV (Fig. 4B and E), which is in line with IHC data and further corroborates the association of MN infection with AFP.

**FIG 5:**
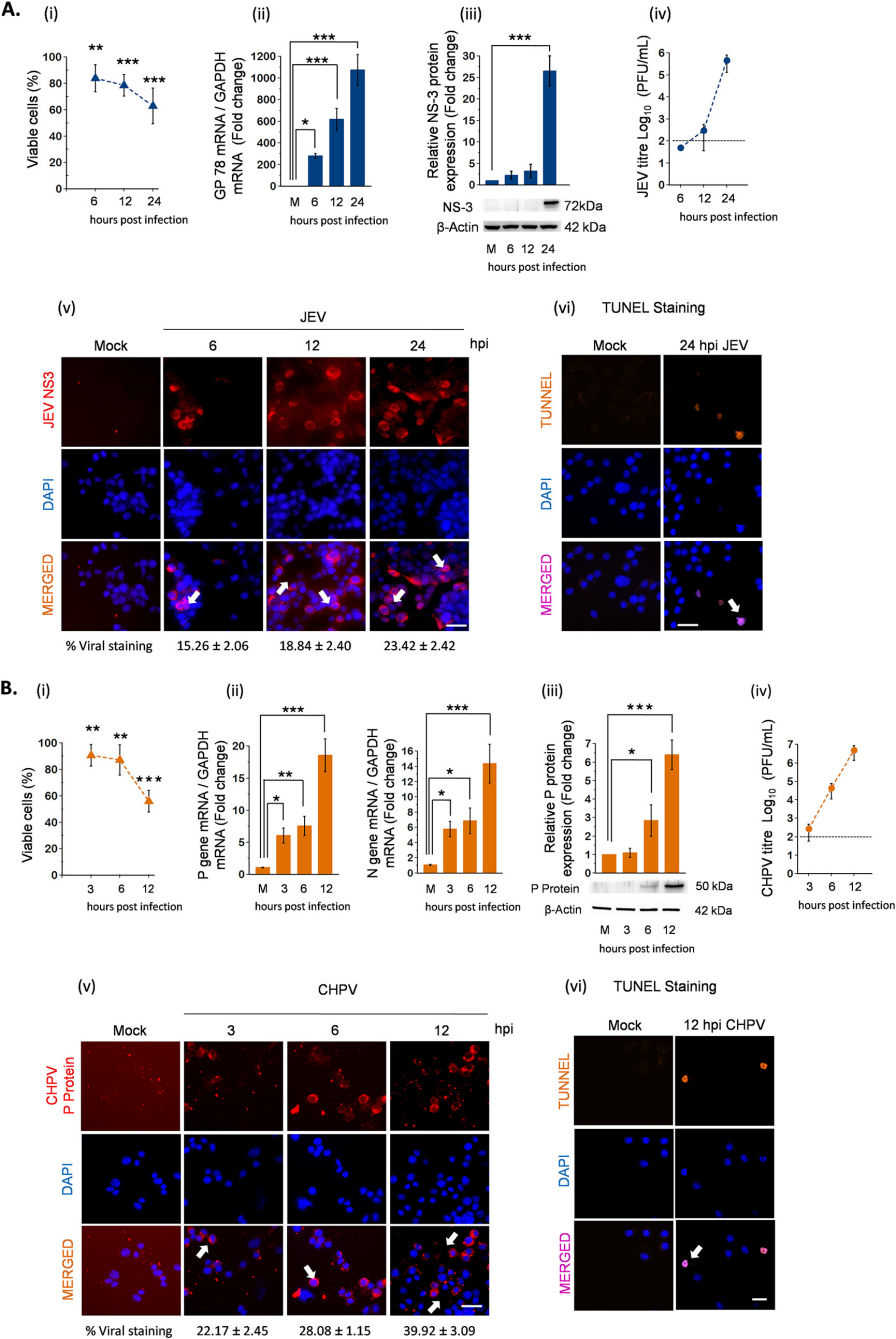
JEV and CHPV replicates actively in motor neuron cell line NSC34. Cells were either left untreated or were infected with JEV at an MOI of 1 and CHPV at an MOI of 0.1 for various times. Blue bar charts represent JEV infection data and orange bar charts represent CHPV infection data. (A and B, i) Percent cell viability of NSC34 cells after JEV and CHPV infection quantified at various time points using WTS assay. (ii) Total RNA was extracted from both mock and experimental NSC34 cells following JEV and CHPV infection at the indicated time points. Relative abundance of viral transcripts was determined using qRT-PCR analysis as fold change, where GAPDH was used as the loading control. (iii) Cell lysates prepared from both mock-infected and virus-infected NSC34 cells were subjected to Western blot analysis at the indicated time points for studying viral protein kinetics. Densitometric analysis was performed with β-actin as a loading control to determine fold change expression of indicated proteins. (iv) Viral titer in NSC34 culture supernatant was determined using plaque assay at indicated time points after JEV and CHPV infection. Dashed lines represent the limit of detection of plaque assays for all experiments conducted. (v) Representative immunostaining of NSC34 cells with antibodies against viral protein NS3 (JEV) and P-protein (CHPV) with nuclear marker DAPI. White arrows show populations of infected NSC34 cells. Scale bar denotes 100 μm. (vi) Representative TUNEL staining of JEV and CHPV at late time points of infection with DAPI stained in blue. Scale bar denotes 100 μm. Here, data represent mean ± SD from a minimum of 3 independent experiments, where statistical significance (*, *P* < 0.05; **, *P* < 0.01; ***, *P* < 0.001; NS, nonsignificant) was calculated using one-way ANOVA followed by *post hoc* Bonferroni correction.

Having shown that viral replication in MN is associated with onset of paralysis, we next studied permissiveness of virus in NSC34 cells to further investigate molecular events contributing to AFP pathogenesis. For this, we infected NSC34 cells with JEV and CHPV at a multiplicity of infection (MOI) of 1 and 0.1, respectively, and harvested samples at various hours postinfection (hpi) to study time-dependent kinetics of viral replication. Phase-contrast images were captured for monitoring cellular health through brief periods of infection, where NSC34 cells displayed cytopathic changes characterized by rounding up of cells by 12 and 24 hpi for JEV and 6 and 12 hpi for CHPV (see Fig. S1 in the supplemental material). To confirm active replication of virus in NSC34 cells, we performed immunostaining with virus-specific antibodies. No signal was detected in mock-infected cells, while with progression of infection, increased viral staining was observed both in JEV (Fig. 5A, v)- and CHPV (Fig. 5B, v)-infected NSC34 cells. In addition to this, terminal deoxynucleotidyltransferase-mediated dUTP-biotin nick end labeling (TUNEL)-positive cells were also observed at 24 hpi for JEV (Fig. 5A, vi) and 12 hpi for CHPV (Fig. 5B, vi), suggesting a correlation between apoptotic death of MN and flaccid paralysis.

10.1128/mBio.02712-21.1 FIG S1 Morphological changes in NSC34 cells upon JEV and CHPV infection. Phase-contrast microscopy images show morphological changes in NSC34 cells after JEV and CHPV infection at different time points of infection. Data are representative of a minimum of three independent experiments performed. Scale bar, 50 μm. Download FIG S1, TIF file, 1.7 MB. Copyright © 2021 Bhaskar et al. 2021 Bhaskar et al. https://creativecommons.org/licenses/by/4.0/ This content is distributed under the terms of the Creative Commons Attribution 4.0 International license.

Next, to study time-dependent kinetics of viral replication, we performed qRT-PCR analysis at various hpi. Significant upregulation of viral copies was evident at 6, 12, and 24 hpi (Fig. 5A, ii), an observation in parallel with time-dependent reduction of viability from 100% in control to 63.08% in 24-h JEV-infected NSC34 cells (Fig. 5A, i). In contrast, infection with CHPV reduced NSC34 viability from 100% to 56% (Fig. 5B, ii) within 12 hpi, concomitant with increased amounts of viral transcripts visible by 3, 6, and 12 hpi (Fig. 5B, ii). Infectious viral particles were detected readily in supernatants obtained upon infection from both viruses studied (Fig. 5A and B, iv) with an increased expression of viral protein NS3 in JEV- and P in CHPV-infected cells (Fig. 5A and B, iii). Additionally, cytokine bead array (CBA) analysis of culture supernatant obtained upon infection further substantiated that seeded NSC34 cells were dying due to active replication of virus and not because of neuroinflammation (Fig. S2).

10.1128/mBio.02712-21.2 FIG S2 Cytokine analysis in NSC34 culture supernatant on viral infection. Cytokine profile of both mock-infected and virus-infected NSC34 cells was studied using cytokine bead array (CBA) at 24 hpi for JEV and 12 hpi for CHPV. Data are represented as mean ± SD from a minimum of 3 independent experiments, where statistical significance (NS, nonsignificant) was calculated using two-tailed paired *t* test. Download FIG S2, TIF file, 1.5 MB. Copyright © 2021 Bhaskar et al. 2021 Bhaskar et al. https://creativecommons.org/licenses/by/4.0/ This content is distributed under the terms of the Creative Commons Attribution 4.0 International license.

### JEV and CHPV infection induces caspase-dependent extrinsic apoptosis in MN.

**FIG 6:**
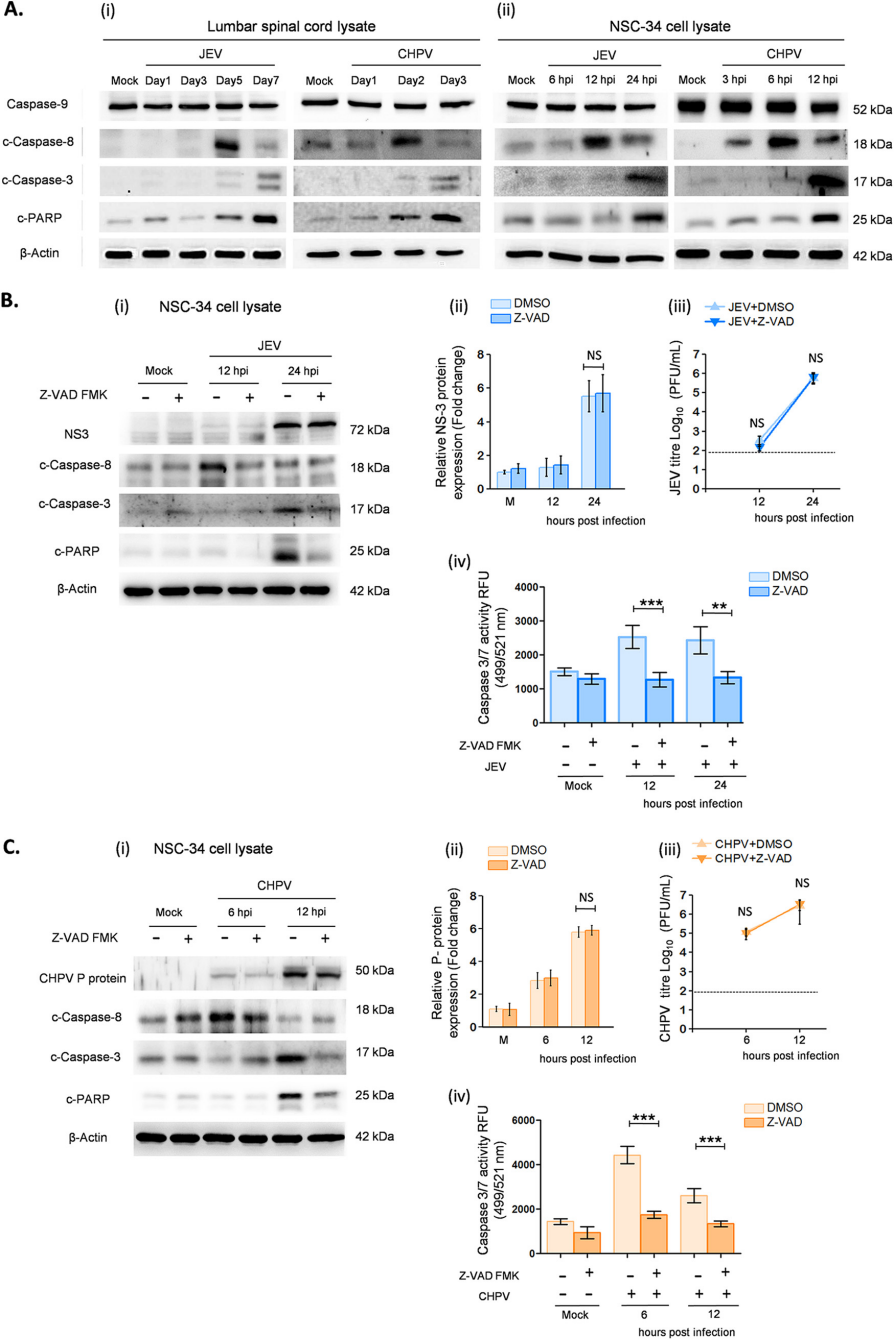
JEV and CHPV infection induces extrinsic apoptosis of motor neurons. For *in vivo* experiments, 10-day-old BALB/c mice were either mock infected or inoculated intraperitoneally with 3 × 10^4^ PFU of JEV or 1 × 10^4^ PFU of CHPV, and for *in vitro* experiments, NSC34 cells were either left untreated or were infected with JEV at an MOI of 1 and CHPV at an MOI of 0.1 for various times as stated previously. Blue bar charts represent data from JEV-infected samples and orange bar charts represent data from CHPV-infected samples. (A, i and ii) Lysates prepared from both lumbar cord tissue and NSC34 cells were subjected to Western blot analysis for studying expression of various active caspases and c-PARP, with β-actin used as a loading control. (B and C, i) NSC34 cells were either pretreated with Z-VAD-FMK solvent (DMSO) or Z-VAD-FMK, a pancaspase inhibitor, for 1 h, followed by incubation with virus at said MOIs. Lysates prepared from both mock-infected and virus-infected NSC34 cells with or without Z-VAD-FMK/DMSO treatment were subjected to immunoblot analysis for studying expression of active caspases and c-PARP. (B and C, ii) Densitometric analysis was performed with β-actin as a loading control to determine fold change expression of viral proteins (NS3 for JEV and P-protein for CHPV) in Z-VAD-FMK or DMSO-treated, mock-infected, or virus-infected NSC34 cells. (B and C, iii) Viral titer was determined in culture supernatant using plaque assay at indicated time points after JEV and CHPV infection in Z-VAD-FMK- or DMSO-treated NSC34 cells. Here, dashed lines represent the limit of detection of plaque assay for all experiments conducted. (B and C, iv) Caspase 3/7 activity was measured in NSC34 cells after JEV and CHPV infection at the indicated time points with or without pretreatment of Z-VAD-FMK/DMSO. Data here represent mean ± SD from a minimum of 3 independent experiments, where statistical significance (*, *P* < 0.05; **, *P* < 0.01; ***, *P* < 0.001; NS, nonsignificant) was calculated using one-way ANOVA followed by *post hoc* Bonferroni correction.

Having shown that viral replication leads to apoptosis *in vitro*, we next wanted to determine which apoptotic pathway is activated in dying MNs. For this, we examined expression levels of various caspases, including caspase-8, caspase-9, and caspase-3, with their associated proteins BAX, BCL2, and c-PARP postinfection. Both *in vivo* and *in vitro* studies revealed significant overexpression of cleaved (c)-casp-8, c-casp-3, and c-PARP protein with no active form of caspase-9 being detected. Distinct upregulation of c-casp8 was observed in NSC34 cells at 12 hpi for JEV and 6 hpi for CHPV, which later declined significantly at 24 hpi for JEV and 12 hpi for CHPV (Fig. 6A, ii). Interestingly, a similar pattern of c-casp8 was observed *in vivo* at day 5 pi for JEV and day 2 pi for CHPV. In addition to this, the active form of casp-3 was detected at 24 hpi *in vitro* and day 7 pi *in vivo* upon JEV and 12 hpi *in vitro* and day 3 pi *in vivo* upon CHPV treatment (Fig. 6A). Further, to validate that MNs were dying via caspase-dependent pathways only, we performed experiments with Z-VAD-FMK, a pancaspase inhibitor. Pretreatment with Z-VAD-FMK attenuated expression levels of cleaved caspases and significantly reduced apoptosis in NSC34 cells compared with respective virus-infected sample (Fig. 6B and C, i). Cytosolic casp3/7 activity was greatly reduced in Z-VAD-treated cells compared with untreated samples (Fig. 6B and C, iv). Plaque assays further confirmed that reduced c-casp-3 in Z-VAD-FMK-treated cells was purely due to inhibition of apoptosis and not because of compromised viral replication (Fig. 6B and C, ii and iii).

### JEV and CHPV infection activates RIG-I pathway.

**FIG 7:**
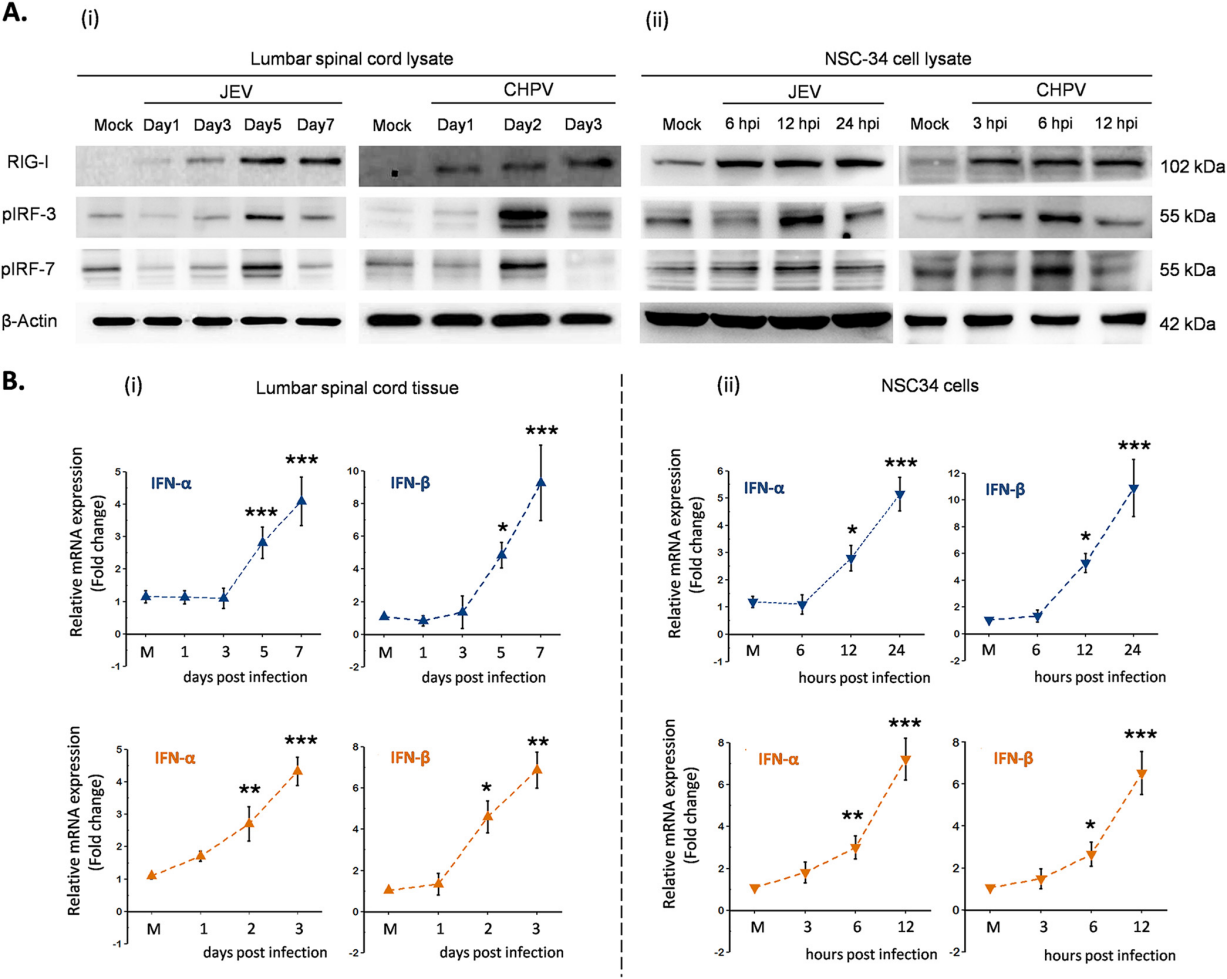
JEV and CHPV activates type-1 interferon production through RIG-I dependent pathway. For *in vivo* studies, 10-day-old BALB/c mice were either mock infected or inoculated intraperitoneally with 3 × 10^4^ PFU of JEV or 1 × 10^4^ PFU of CHPV, and for *in vitro* paradigms, NSC34 cells were either left uninfected or were infected with JEV at an MOI of 1 and CHPV at an MOI of 0.1 for various time points of infection. Blue bar charts represent data from JEV-infected samples and orange bar charts represent data from CHPV-infected samples. (A, i and ii) Lysates prepared from both lumbar cord tissue and NSC34 cells were subjected to Western blot analysis for studying expression of RIG-I and downstream proteins, with β-actin used as a loading control for all experiments. (B, i and ii) Total RNA was extracted from both lumbar spinal cord and NSC34 cells for studying gene expression of type-1 IFNs using qRT-PCR, with fold change being calculated with GAPDH as a loading control. For all experiments, data represent mean ± SD from a minimum of 3 independent experiments, where statistical significance (*, *P* < 0.05; **, *P* < 0.01; ***, *P* < 0.001; NS, nonsignificant) was calculated using one-way ANOVA followed by *post hoc* Bonferroni correction.

Next, we studied expression level of RIG-I, a well-described PRR involved in initiating antiviral signaling upon infection, both in infected lumbar tissue and NSC34 cells. Immunoblot analysis showed significant upregulation of RIG-I at days 5 and 7 pi for JEV and days 2 and 3 pi for CHPV in mice, with similar trends in infected NSC34 cells, where marked upregulation of RIG-I was evident by 6 hpi for JEV and 3 hpi for CHPV (Fig. 7A, i and ii). A transient upregulation of phosphorylated IRF3 and pIRF7, critical markers of the RIG-I/interferon signaling axis, was detected at 5 dpi for JEV and 2 dpi for CHPV that eventually declined at later days of infection. Consistent with our *in vivo* data, infected NSC34 cells showed similar trends of pIRF3 and pIRF7 at 12 hpi for JEV and 6 hpi for CHPV that declined over time. Activation of type 1 interferon signaling was confirmed by qRT-PCR analysis of IFN-α and IFN-β, both in infected lumbar tissue and NSC34 cells by day 5 pi *in vivo* and 6 hpi *in vitro* upon JEV and day 2 pi *in vivo* and 3 hpi *in vitro* upon CHPV (Fig. 7B, i and ii).

**FIG 8:**
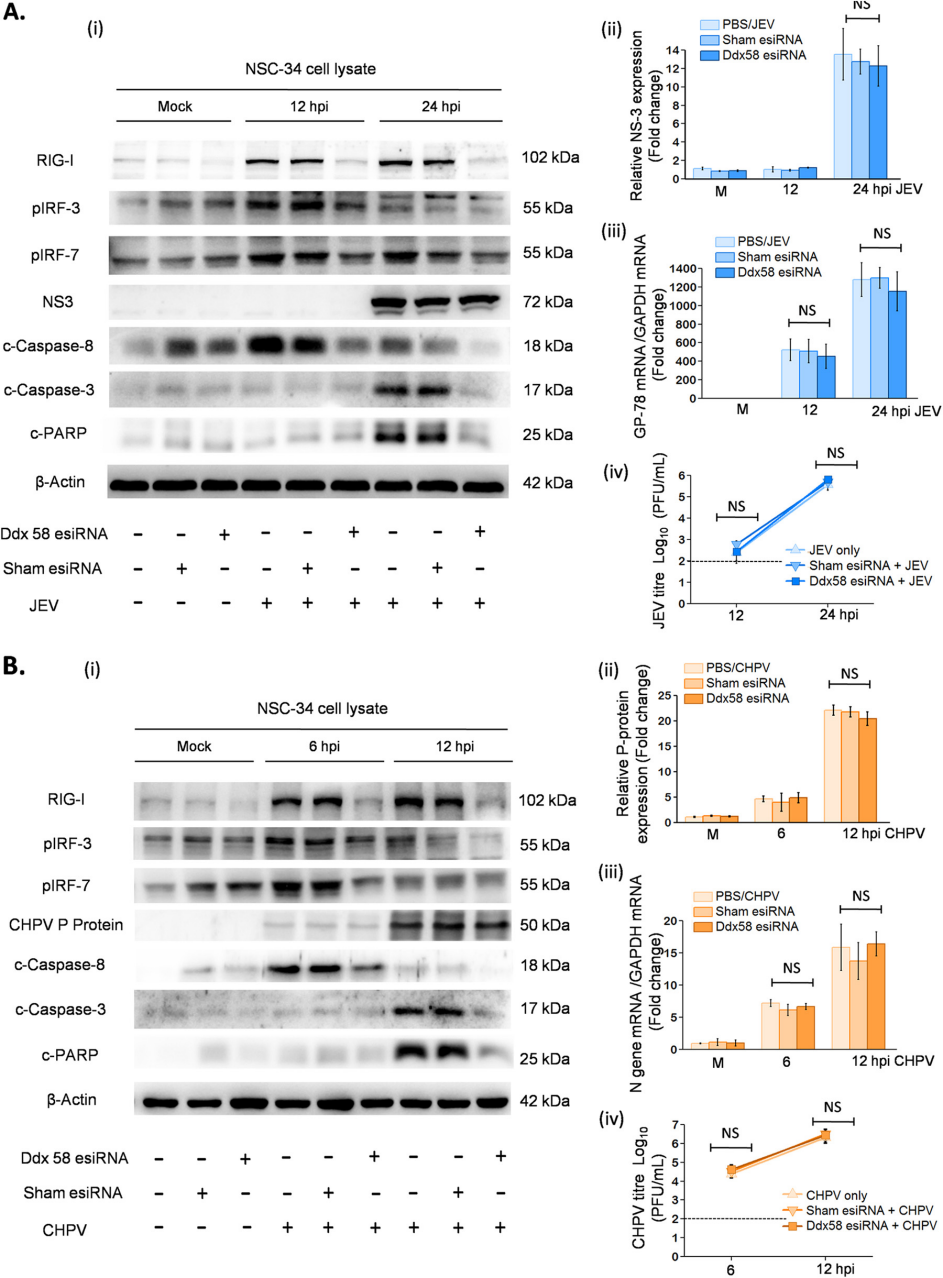
RNA interference using specific RIG-I siRNA abrogates MN apoptosis *in vitro*. NSC34 cells were either transfected with specific RIG-I esiRNA or negative-control eGFP esiRNA for 8 h. Posttransfection, cells were maintained in DMEM for another 16 h and were later infected with JEV at an MOI of 1 and CHPV at an MOI of 0.1 for indicated time points. (A and B, i) Cell lysates prepared from mock-infected control, only virus-infected NSC34, mock-infected RIG-I or eGFP-transfected cells, and virus-infected RIG-I or eGFP-transfected cells were subjected to immunoblot analysis for studying protein expression of RIG-I, pIRF3, pIRF7, NS3, P protein, cleaved caspases, cleaved-PARP, and β-actin. (A and B, ii) Densitometric analysis was performed with β-actin as a loading control to determine fold change in expression of viral proteins (NS3 for JEV and P-protein for CHPV) in mock-infected or virus-infected NSC34 cells with or without transfection with RIG-I siRNA or negative-control eGFP siRNA. (A and B, iii) Total RNA was extracted from both mock and experimental NSC34 cells following JEV and CHPV infection posttransfection at the indicated time points. Relative abundance of viral transcripts was determined using qRT-PCR analysis as fold change, where GAPDH was used as a loading control. (A and B, iv) Plaque assays were conducted using NSC34 culture supernatant after RIG-I-specific or negative-control eGFP transfection, followed by JEV and CHPV infection at the indicated time points. In the bar graph, dashed lines represent the limit of detection of plaque assay for experiments performed. Data represent means ± SD from a minimum of 3 independent experiments, where statistical significance (*, *P* < 0.05; **, *P* < 0.01; ***, *P* < 0.001; NS, nonsignificant) was calculated using one-way ANOVA followed by *post hoc* Bonferroni test.

Thereafter, we employed short interfering RNA (siRNA)-mediated silencing to knock down RIG-I expression to validate its role in virus-induced caspase-dependent death of MNs. Gene silencing using specific RIG-I (Ddx58) esiRNA significantly downregulated cleaved caspases and rescued apoptosis at 12 and 24 hpi for JEV and 6 and 12 hpi for CHPV (Fig. 8A and B) with survival of live-NSC34 cells (Fig. S5). However, no changes in viral protein expression (Fig. 8A and B, ii) were detected, indicating that downregulation of apoptotic effector proteins upon gene silencing were not due to impaired viral replication. In addition to this, we performed qRT-PCR analysis and plaque assays to further substantiate our findings, which, in parallel to our observations, showed no significant differences in viral replication (Fig. 8A and B, iii and iv). There was significant decrease in pIRF3 and pIRF7 expression at 12 hpi for JEV and 6 hpi for CHPV in RIG-I-transfected NSC34 compared with the respective negative control. In line with immunoblot analysis, siRNA-mediated RIG-I silencing downregulated type 1 interferon production in both JEV- and CHPV-infected cells (Fig. S3), indicating that upon infection both viruses activate the RIG-I/pIRF3-7/interferon axis to initiate virus-induced apoptosis.

10.1128/mBio.02712-21.3 FIG S3 Relative type-1 interferon expression in RIG-I-depleted NSC34 cells upon JEV and CHPV infection. Total RNA was extracted from mock-infected control, virus-infected NSC34, mock-infected RIG-I, or eGFP-transfected NSC34 and virus-infected RIG-I or eGFP-transfected NSC34 cells for studying type-1 IFN expression using qRT-PCR, with fold change being calculated with GAPDH as a loading control. Data are represented as mean ± SD from a minimum of 3 independent experiments, where statistical significance (*, *P* < 0.05; **, *P* < 0.01; ***, *P* < 0.001; NS, nonsignificant) was calculated using one-way ANOVA followed by *post hoc* Bonferroni correction. Download FIG S3, TIF file, 1.3 MB. Copyright © 2021 Bhaskar et al. 2021 Bhaskar et al. https://creativecommons.org/licenses/by/4.0/ This content is distributed under the terms of the Creative Commons Attribution 4.0 International license.

10.1128/mBio.02712-21.5 FIG S5 Percent live/dead population in RIG-I-transfected NSC34 versus negative-control (NC) transfected NSC34 upon virus infection. Cells were either transfected with RIG-I specific siRNA (Ddx58) or negative-control (NC) siRNA (eGFP) and were infected with JEV at an MOI of 1 and CHPV at an MOI of 0.1 for various time points postinfection. Green bars represent live NSC34 population, and red bars represent dead NSC34 population. Data plotted as percent live population after JEV and CHPV infection at various time points and are a representation of a minimum of 3 independent experiments (mean ± SD) performed, where statistical significance (*, *P* < 0.05; **, *P* < 0.01; ***, *P* < 0.001; NS, nonsignificant) was calculated using one-way ANOVA followed by *post hoc* Bonferroni correction. Download FIG S5, TIF file, 1.1 MB. Copyright © 2021 Bhaskar et al. 2021 Bhaskar et al. https://creativecommons.org/licenses/by/4.0/ This content is distributed under the terms of the Creative Commons Attribution 4.0 International license.

### RIG-I-mediated apoptosis is independent of type 1 interferon pathway.

**FIG 9:**
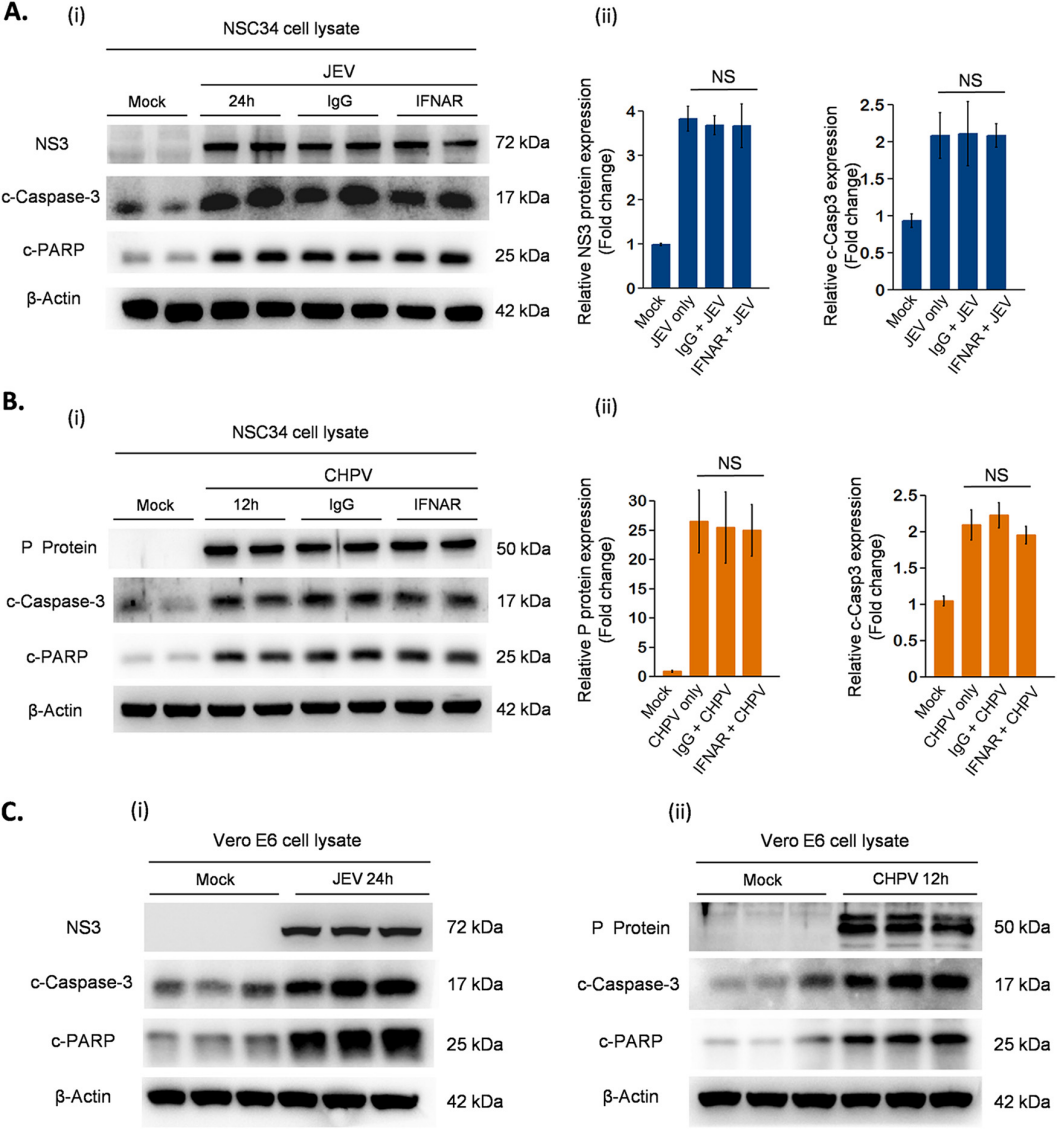
Characterization of mechanism involved in virus-induced apoptosis of NSC34 cells. Experiments were performed with IFN-responsive cell line NSC34 and IFN-unresponsive cell line Vero E6. NSC34 cells prior to infection were treated with neutralizing antibodies against IFNAR receptor or IgG control for 1 h, followed by infection with either JEV or CHPV at said MOIs. Postinfection, cells were maintained again with neutralizing antibody IFNAR or IgG control at a concentration of 10 μg/ml. Similar to NSC34, E6 Vero cells were mock infected and infected with JEV at an MOI of 1 and CHPV at an MOI of 0.1 for 24 and 12 h, respectively. (A, B, and C) Cell lysate prepared from both NSC34 cells and Vero E6 cells postinfection was subjected to Western blot analysis for studying expression of viral protein, cleaved-casp-3, and c-PARP, with β-actin as a loading control. (A and B, ii) Densitometric analysis was performed using FIJI to determine fold change in expression of viral proteins and cleaved-casp-3 in mock-infected or virus-infected NSC34 cells with or without treatment with IFNAR or IgG control antibody. The data are represented as mean ± SD from a minimum of 3 independent experiments, where *P* values were calculated (*, *P* < 0.05; **, *P* < 0.01; ***, *P* < 0.001) using one-way ANOVA followed by *post hoc* Bonferroni correction.

Having shown that siRNA-mediated RIG-I silencing downregulated IFN expression and rescued MN death, we next determined the direct role of IFN signaling in virus-induced apoptosis. To address this, we performed two experiments, first with interferon-responsive cell line NSC34, where the receptor antibody neutralization assay was carried out to block any downstream IFN signaling, and second with the interferon-unresponsive cell line Vero. Surprisingly, in both experiments, JEV and CHPV led to apoptosis of NSC34 and Vero cells with distinct upregulation of c-casp3 and c-PARP proteins (Fig. 9A to C). NSC34 cells upon infection displayed no significant changes in the active form of casp-3 and viral protein expression for all three conditions studied (Fig. 9A and B, ii). Similar to NSC34 cells, Vero cells displayed cytopathic changes like rounding up of cells and refraction upon infection (data not shown), suggesting that both viruses induce apoptosis independent of host IFN signaling.

### RIG-I deficiency suppresses viral replication *in vivo*.

**FIG 10:**
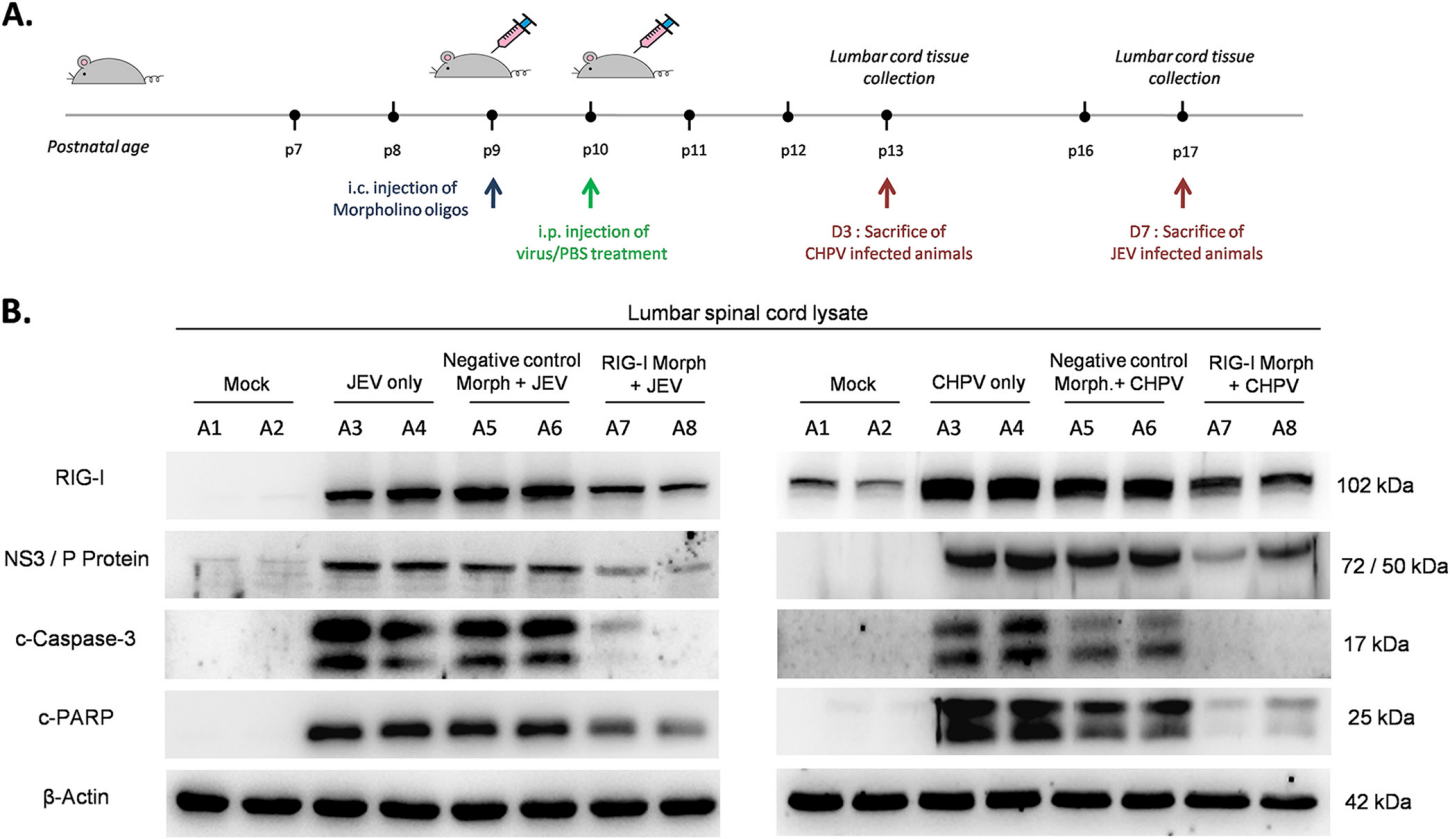
Inhibition of RIG-I *in vivo* abrogates cellular apoptosis in lumbar cord tissue. (A) Nine-day-old BALB/c mice were either treated with RIG-I-specific vivo-morpholino or negative-control vivo-morpholino (mock-morpholino) intracranially. After 24 h, pups were either injected with PBS (mock infection) or JEV at an MOI of 3 × 10^4^ PFU or CHPV at an MOI of 1 × 10^4^ PFU intraperitoneally. Lumbar cord tissue was harvested at day 7 pi for JEV and day 3 pi for CHPV, when pups displayed complete hindlimb paralysis with difficult or no movement at all. (B) Tissue lysates prepared from both mock-infected and virus-infected pups with or without specific vivo-morpholino or negative-control vivo-morpholino treatments were subjected to immunoblot analysis for studying expression of RIG-I receptor, viral proteins (NS3 for JEV and P-protein for CHPV infection), cleaved-casp-3, and cleaved-PARP, with β-actin used as a loading control. Data here are a representation of a minimum of 3 independent experiments performed.

To explore whether RIG-I knockdown in mouse SC altered the behavioral outcome of flaccid paralysis and whether such changes were associated with reduction in virus-induced apoptosis, we infected RIG-I morpholino-treated (RIG-I-Mo) BALB/c mice with virus (Fig. 10A). Immunoblot analysis from RIG-I-Mo mice showed effective RIG-I silencing compared with negative control-treated morpholino (NC-Mo) and only virus-infected animals (Fig. 10B). Significant upregulation of c-casp3 and c-PARP was detected in both only virus-infected (A3, A4) and infected NC-Mo mice (A5, A6), while a lumbar isolate of infected RIG-I-Mo (A7, A8) animals showed drastic reduction in c-casp3 and c-PARP expression. Notably, both JEV and CHPV-infected RIG-I-Mo mice displayed remarkable reduction in viral protein expression compared with respective NC-Mo and only infected animals, suggesting an important role of the RIG-I/pIRF3-7 axis in viral apoptosis and replication *in vivo*.

## DISCUSSION

In this study, we propose an AFP mouse model with detailed characterization of molecular pathways that lead to paralysis and spinal MN death. Here, we have used clinical isolates of two different neurotropic viruses belonging to two different families, namely, *Flaviviridae* and *Rhabdoviridae*, for inducing paralysis in mice. When injected intraperitoneally, both viruses successfully induced AFP with consistent features of abnormal posture, hindlimb clasping, and gait abnormalities. This observation is in line with retrospective studies performed with JE-infected patients who presented prodromal features of nonspecific illness, which rapidly progressed to flaccid paralysis both in JE conscious and convalescent patients (41, 42).

Since lower-limb paralysis is a more commonly reported symptom of neurotropic virus infections, we mainly restricted our focus to the lumbar segment of SC. Both JEV- and CHPV-infected lumbar sections displayed features of chromatolysis, with increased viral replication in infected tissue with time. Consistent with these findings, paralyzed mice showed direct colocalization of SMI32 with virus-specific antigens, suggesting a strong association between paralysis onset and virus replication. Additional support came from studies with JE-conscious patients who displayed damaged anterior horn cells on electrophysiological and MRI experiments (27, 43). Since it is difficult to delineate complex signaling pathways leading to MN death in animal models, we planned to perform experiments employing a reductionist model comprised of infection of motor neuron cell line NSC34 with JEV and CHPV. Together, findings from our *in vivo* and *in vitro* experiments led us to conclude that direct viral injury is responsible for paralytic disease and MN death upon infection.

Numerous reports previously demonstrated the role of apoptosis in pathogenesis of lethal encephalitis (30, 31, 34, 44); however, no report has yet illustrated the role of spinal MN death in virus-induced AFP. In this study, we reported conclusively that upon infection MN undergoes extrinsic apoptotic signaling via activation of the RIG-I/pIRF3-7 axis. Essentially, we demonstrated that RIG-I-transfected-NSC34 cells abrogated apoptosis, with no significant activation of other viral PAMP expression (see Fig. S4 in the supplemental material), suggesting that RIG-I, not the TLR system, plays a critical role in MN apoptosis. Interestingly, RIG-I ablation in NSC34 cells reduced interferon expression upon infection, reinforcing the notion that IFN signaling is activating virus-induced apoptosis of MNs. However, receptor-blocking experiments with IFNAR antibody clearly suggested that virus-induced apoptosis in NSC34 cells is independent of interferon signaling. It is possible that RIG-I activation stimulates the production of pIRF3-induced IFN-independent genes (likely ISG54 and ISG56) that eventually lead to infection-elicited apoptosis (45, 46). Nonetheless, it needs to be highlighted that it is still uncertain how IFN-independent genes orchestrate the induction of apoptosis during infection in MNs.

10.1128/mBio.02712-21.4 FIG S4 Studying relative expression of PAMP MDA5, TLR3, TLR4, and TLR7 upon infection in RIG-I-depleted NASC34 cells. Protein lysates prepared from positive-control, mock-infected control, virus-infected NSC34, mock-infected RIG-I, or eGFP-transfected cells and virus-infected RIG-I or eGFP-transfected NSC34 cells were subjected to immunoblot analysis for studying expression of viral protein and various PAMPs, including MDA5, TLR3, TLR4, and TLR7, with β-actin as a loading control. To rule out the possibility of an experimental error in probing blots, positive control samples were included for each PAMP in respective gels. Lysate prepared from the AGS cell line was used as a positive control for MDA5. Similarly, JEV-infected N2A cells at 36 hpi were used as a positive control for TLR3 and TLR7, whereas lysate prepared from JEV-infected N9 cells (36 hpi) served as a positive control for TLR4. Data here are a representation of 3 independent experiments. Download FIG S4, TIF file, 0.7 MB. Copyright © 2021 Bhaskar et al. 2021 Bhaskar et al. https://creativecommons.org/licenses/by/4.0/ This content is distributed under the terms of the Creative Commons Attribution 4.0 International license.

Surprisingly, with our *in vivo* model, we made contrasting observations where CNS ablation of RIG-I significantly enhanced resistance to infection, clearly visible by reduced expression of viral proteins and apoptotic markers rather than increased susceptibility to infection, as reported previously (46, 47). Notably, in our paradigm, transient ablation of RIG-I in CNS using vivo-morpholino attenuated both antiviral and inflammatory arms of innate immune response, which may have interfered with replication of virus in SC compared to the control-treated morpholino animals, which displayed no change in virus protein expression. A plausible explanation for our interesting *in vivo* observation could be the decreased permeability of the BBB for systemic virus due to localized ablation of RIG-I in brain and SC. Although our *in vivo* data do not strongly support whether attenuated cellular death in RIG-I-knockdown animals is mediated directly via type 1 interferon response and/or indirectly via host cytokine response upon infection, our results provide one clear explanation as to how transient silencing of RIG-I in motor neuron NSC34 cells attenuated apoptosis by suppressing pIRF3/7 signaling effectively by blocking p-IRF3-induced IFN-independent genes (likely ISG54 and ISG56) and not any other IFN-dependent pathways (interferon-β production and IFNAR signaling).

In conclusion, the central finding of this paper is that upon infection, MNs induce RIG-I-dependent activation of apoptosis and type 1 interferon pathways, although upon infection the host cell remains resistant to antiviral effects of IFNs and undergoes apoptosis by following IFN-independent pathways.

## MATERIALS AND METHODS

### Viruses.

The GP78 strain of JEV was propagated in suckling BALB/c mice (48), while CHPV strain 1653514, a kind gift by Dhrubajyoti Chattopadhyay (Amity University, Kolkata, India), was propagated in the Vero E6 cell line (35). Both viral preparations were analyzed briefly for determining number of PFU using plaque assay as described earlier (49).

### Mouse experiments and behavioral scoring.

All studies performed with animals were approved by the Animal Ethics Committee of National Brain Research Centre (approval no. NBRC/IAEC/2017/130) and were in accordance with the guidelines of the Committee for the Purpose of Control and Supervision of Experiments on Animals (CPCSEA), Ministry of Environment and Forestry, Government of India. BALB/c mice were purchased from The Jackson Laboratory (Bar Harbor, ME) and were housed at the pathogen-free and climate-controlled animal facility of the NBRC. Viral infections were performed on 10-day-old litters that were always housed with their mothers for milk feeding. Pups of either sex were inoculated intraperitoneally with 3 × 10^4^ PFU of JEV or 10^4^ PFU of CHPV in a 25-μl volume. Mice were examined twice daily, and behavioral experiments were performed once a day until clinical features of paralysis appeared (Table 1).

**TABLE 1:** Behavioral assessment and animal scoring after viral infection

Test type	Description
General locomotory behavior test^*a*^ (GLB score)	0, normal locomotion supported by all four limbs; abdomen not touching the ground
	1, tip-toe walk with elevated abdomen
	2, hind leg inhibition with severe limp and lowered pelvis
	3, difficult or no movement, paralysis of hindlimbs and abdomen touching the ground
Hindlimb clasping test^*b*^ (HLC score)	0, hindlimbs consistently splayed away from midline
	1, one of the hindlimb retracted towards midline
	2, both hindlimbs partially retracted towards midline for more than 50% of observation time
	3, both hindlimbs completely retracted towards midline for more than 50% of suspended time
Footprinting test^*c*^	Each run was analyzed for three parameters: stride length, sway length, and stance length (distance in cm)

a For evaluating GLB score, experimental pups were allowed to move freely in an open box having dimensions of 35 by 15 cm and were scored 0, 1, 2, and 3, representing none, moderate, marked, and extreme magnitude of locomotion deficits.

b For evaluating HLC score, mice were grasped from the base of the tail and suspended briefly in the air for 15 s, and pups were scored 0, 1, 2, and 3 on the basis of the position of hindlimbs.

c For the footprinting test, hind paws of mice were painted in nontoxic dye and pups were motivated to walk on narrow lanes lined with white paper. For analysis and quantification, the first few and last few footmarks were excluded and only the middle portion of each run was evaluated.

### Tissue processing and IHC.

For lumbar cord isolation, pups were deeply anaesthetized with ketamine and perfused transcardially using phosphate-buffered saline (PBS). Mice were sacrificed at days 1, 3, 5, and 7 of JEV and days 1, 2, and 3 of CHPV infection. Tissue samples obtained were used for RNA and protein isolation; however, for immunohistochemistry (IHC) and Nissl staining, pups were transcardially fixed with paraformaldehyde (PFA) solution. Isolated lumbar cords were postfixed in PFA overnight, followed by cryopreservation in 30% sucrose until saturated. Serial sections of 20-μm thickness were prepared using a cryostat (Leica CM3050S) and permeabilized with 0.1% Triton X-100 in PBS. Afterwards, sections were blocked with serum and incubated overnight with primary antibodies NS3 (1:500; GeneTex), CHPV P protein (1:500; kind gift by Bharat Biotech International Limited, Hyderabad, India), and SMI-32 (1:500; Millipore) at 4°C. Stained sections next were subjected to PBXT washes and were later incubated with appropriate Alexa-fluor-tagged secondary antibody. Tissue sections were then either double stained using second primary antibody or mounted with 4′,6-diamidino-2-phenylindole (DAPI) (Vector Laboratories, USA). Images were captured on a Zeiss Apotome microscope (Carl Zeiss, Germany) at ×20 magnification.

### Nissl staining.

Lumbar sections were briefly rehydrated in Milli-Q and were subjected to cresyl violet staining. Sections were next immersed in differentiating solution (1:1 absolute ethanol and dioxane), followed by dehydration in dioxane (Merck-Millipore, Sigma) and tissue clearance in xylene (Merck-Millipore, Sigma). Finally, slides were mounted with DPX (dibutylpthalate polystere xylene) (Sigma) and were dried for 24 h in dark boxes. Images were captured on a Leica DMRXA2 microscope at ×5 and ×40 oil magnifications.

### Cell lines, infections, and Z-VAD-FMK treatment.

Motor neuron cell line NSC34, a kind gift from Jean-Pierre Julien (Laval University, Canada), Vero E6 cell line, a gift from Debi P. Sarkar (Delhi University, India), and porcine stable kidney cells (PS cells), a gift from G. R. Medigeshi (THSTI, India), were cultured in Dulbecco’s modified Eagle’s medium (DMEM) (Gibco), while a gastric adenocarcinoma cell line (AGS), a kind gift from Ellora Sen, was cultured in DMEM F-12 supplemented with 10% fetal bovine serum (FBS), penicillin (100 U/ml), and streptomycin (100 mg/ml) (Gibco) at 37°C. NSC34 cells were seeded and switched to serum-free DMEM upon reaching confluence 2 h prior to virus inoculation. For experiments, cells were infected at a multiplicity of infection (MOI) of 1 for JEV or 0.1 for CHPV for 2 h, followed by PBS wash to remove any noninternalized virus. Monolayers were then maintained in 5% DMEM, and cells were harvested at various time points postinfection for time-dependent studies. For Z-VAD-FMK (InvivoGen, USA) inhibitor experiments, NSC34 cells were divided into four groups: mock infection, virus infection, mock infection and Z-VAD-FMK treatment, and virus infection and Z-VAD-FMK treatment. Cells were pretreated with Z-VAD-FMK (20 μM) for 2 h and were later infected with virus. Postinfection, samples were harvested and cell supernatants were collected at the indicated time points for various cell death assays and immunoblotting experiments.

### Cell viability assay.

Viability of cultured cells was determined using cell proliferation reagent WST1 (Roche, Sigma) per the manufacturer’s instructions. NSC34 cells were seeded at a density of 2 × 10^4^ cells per well in separate 96-well plates, followed by infection at said MOIs. Twenty microliters of WST1 solution was added to each well after 6, 12, and 24 hpi for JEV and 3, 6, and 12 hpi for CHPV. Absorbance was recorded at 490 nm using a Multiskan Sky spectrophotometer (Thermo Fischer), reflecting the formation of formazan product by metabolically active cells. Results were expressed as relative percentages of virus-infected cells to that of mock-infected cells.

### Transfection procedures and siRNA knockdown.

Transient transfection of NSC34 cells was performed using Lipofectamine RNAiMAX reagent (Invitrogen, USA) per the manufacturer’s instructions. Briefly, cells were cultured in DMEM until they attained a confluence of 60 to 70%, after which cells were transfected with 30 pmol siRNA specific to RIG-I (Ddx58) and a negative-control enhanced green fluorescent protein (eGFP). Transfection was performed in Opti-MEM for 8 h, after which cells were maintained in 5% DMEM for 16 h. Cells were then serum starved for an additional 2 h before being treated with virus as stated earlier. Postinfection, samples were harvested and supernatants were collected at different time points for various biochemical assays.

### Live/dead assay.

NSC34 cells were briefly seeded in 12-well plates and transfected upon reaching confluence. Posttransfection, cells were treated with virus at said MOIs for 12 and 24 hpi for JEV and 6 and 12 hpi for CHPV. NSC34 cells were then incubated with LIVE/DEAD reagent (Invitrogen, Thermo Fischer) at a concentration of 4 μM ethidium dimer-D1 and 2 μM Calcein-AM in the dark. Images were captured and analyzed using a Zeiss Axio Vert.1 microscope at ×10 magnification.

### Immunocytochemistry.

Briefly, NSC34 cells were grown in 8-well chamber slides, followed by infection with JEV and CHPV at said MOIs. Postinfection, cells were fixed with 4% paraformaldehyde solution and were blocked using bovine serum albumin (Sigma). Cells were then incubated overnight with primary antibodies NS3 (1:500) and CHPV P protein (1:500) at 4°C, followed by extensive washing. Finally, cells were incubated with appropriate Alexa fluor-tagged secondary antibody and were mounted with DAPI. Images were captured on a Zeiss Apotome microscope at ×20 magnification.

### RNA isolation and qRT-PCR analysis.

Total cellular RNA was isolated from lumbar cord tissue and NSC34 cells using Tri reagent (Sigma-Aldrich, USA). Briefly, cDNA was synthesized using random hexamer primers (Verso cDNA synthesis kit), and quantitative real-time PCRs (qRT-PCRs) were performed using Power SYBR green (Applied Biosystems, USA) with gene-specific primers (Table 2). Relative gene abundance for each reaction was determined using the delta threshold cycle method with glyceraldehyde-3-phosphate dehydrogenase (GAPDH) as a loading control in a ViiA 7 real-time PCR system (Applied Biosystems). Results were expressed as fold differences between mock- and virus-infected samples.

**TABLE 2:** Gene-specific primers

Primer	Sequence
GP-78	5′-TTGACAATCATGGCAAAC-3′ (sense)
	5′-CCCAACTTGCGCTGAATAA-3′ (antisense)
CHPV P gene	5′-ACCTGGCTCCAAATCCAATAC-3′(sense)
	5′-GGTGGATCAGACGGAGAGATA-3′ (antisense)
CHPV N gene	5′-CATTGTCCACTCTGTCACAC-3′ (sense)
	5′-GGCATGTAGGAATCAGCT-3′ (antisense)
GAPDH	TCTCCCTCACAATTTCCATCC (sense)
	GGGTGCAGCGAACTTTATTG (antisense)
IFN-α	5′-ATTGGCTAGGCTCTGTGCTTT-3′ (sense)
	5′-AGGGCTCTCCAGACTTCTGC-3′ (antisense)
IFN-β	5′-AAGAGTTACACTGCCTTTGCCATC-3′ (sense)
	5′-CACTGTCTGCTGGTGGAGTTCATC-3′ (antisense)

### Protein isolation and immunoblotting.

Whole-cell protein extract and tissue lysates were prepared as described previously (50), with protein concentration estimated using bicinchoninic acid (BCA) reagent. Equal amounts of protein samples were resolved by SDS-PAGE and transferred onto nitrocellulose membrane. Subsequently, membranes were blocked and incubated with primary antibodies specific for RIG-I (1:1,000; CST), cleaved-caspase-3 (1:1,000; CST), c-casp-8 (1:1,000; CST), caspase-9 (1:1,000; CST), cleaved-poly ADP-ribose polymerase (c-PARP; 1:2,000; Abcam), pIRF-3 (1:2,000; CST), pIRF-7 (1:2,000; CST), NS-3 (1:10,000), CHPV P protein (1:3,000), MDA5 (1:1,000; Abcam), TLR3 (1:1,000; Santa Cruz), TLR4 (1:1,000; Abcam), TLR7 (1:1,000; Abcam), and β-actin (1:10,000; Sigma) overnight at 4°C. After extensive washes, blots were probed with peroxidase-conjugated secondary antibodies (Vector Laboratories) and developed using chemiluminescence reagent (Millipore). Images were captured using the Chemigenius bioimaging system (Uvitec Cambridge). To ensure equivalent loading of samples, blots were stripped and reprobed with β-actin.

### Quantification of soluble cytokines using flow cytometry.

Mouse cytokines interleukin-6 (IL-6), interleukin-10 (IL-10), monocyte chemoattractant protein-1 (MCP-1), IFN-γ, tumor necrosis factor (TNF), and interleukin-12p70 (IL-12p70) were estimated in culture supernatants of virus-infected NSC34 using a mouse inflammation CBA kit (BD Biosciences, USA) per the manufacturer’s instructions. Analysis was carried out in FACS Verse (BD Biosciences) using FCAP 2.0 analysis software.

### TUNEL staining.

TUNEL assay was carried out using Promega’s DeadEnd fluorometric TUNEL system per the manufacturer’s protocol. Briefly, NSC34 cells were seeded at a density of 2 × 10^5^ cells per well in 8-well chamber slides and infected with virus as described before. Postinfection, cells were fixed with PFA, followed by incubation with recombinant terminal deoxynucleotidyl transferase (rTdT) enzyme mix. Later, cells were washed extensively and mounted with Vectashield mounting medium and DAPI. Images were captured on a Zeiss Apotome at ×20 magnification.

### Antibody neutralization assay.

For antibody-based neutralization assay, NSC34 cells were seeded at a density of 5 × 10^4^ cells per well on 24-well plates. Prior to infection, cells were treated with 10 μg/ml isotype IgG1 К (clone MOPC1; BioLegend) or purified anti-mouse IFNAR-1 (clone MARI-5A3; BioLegend) blocking antibody for 1 h. Cells were then washed with PBS once and infected with virus at said MOIs. Postinfection, cells were maintained in 5% DMEM containing 10 μg/ml neutralizing antibodies and samples were harvested at the indicated time points for immunoblotting.

### RIG-I gene knockdown and mouse infection.

For vivo-morpholino experiments, 10-day-old BALB/c pups were randomly assigned to four groups: group 1, mock infection; group 2, virus infection only; group 3, virus infection and negative-control vivo-morpholino; and group 4, virus infection and RIG-I vivo-morpholino. Starting a day prior to infection, mice belonging to groups 3 and 4 were injected intracranially with a single dose of RIG-I-specific vivo-morpholino (18.5 mg/kg of body weight; Gene Tools, USA) and negative control (18.5 mg/kg), respectively. After 24 h, all animals were infected intraperitoneally with either PBS or virus at day 0 of infection. Animals were then sacrificed at day 7 pi for JEV and day 3 pi for CHPV, and lumbar cords were harvested for immunoblot analysis.

### Statistical analysis.

Data were represented as means ± standard deviations (SD) from a minimum of three independent experiments unless otherwise stated. GraphPad Prism 5 and KyPlot2.0 were used for data analysis and preparation of graphs. Time-course studies were analyzed using one-way analysis of variance (ANOVA) with Bonferroni *post hoc* test, whereas differences between two groups were evaluated using paired two-tailed Student's *t* test with 95% confidence. Any *P* value less than 0.05 was considered statistically significant.

## References

[1] Solomon T, Kneen R, Dung NM, Khanh VC, Thuy TT, Ha DQ, Day NP, Nisalak A, Vaughn DW, White NJ. 1998. Poliomyelitis-like illness due to Japanese encephalitis virus. Lancet 351:1094–1097. doi:10.1016/S0140-6736(97)07509-0. 9660579

[2] Suresh S, Forgie S, Robinson J. 2018. Non-polio Enterovirus detection with acute flaccid paralysis: a systematic review. J Med Virol 90:3–7. doi:10.1002/jmv.24933. 28857219

[3] Saraswathy TS, Zahrin HN, Apandi MY, Kurup D, Rohani J, Zainah S, Khairullah NS. 2008. Acute flaccid paralysis surveillance: looking beyond the global poliomyelitis eradication initiative. Southeast Asian J Trop Med Public Health 39:1033–1039. 19062691

[4] Johnstone J, Hanna SE, Nicolle LE, Drebot MA, Neupane B, Mahony JB, Loeb MB. 2011. Prognosis of West Nile virus associated acute flaccid paralysis: a case series. J Med Case Rep 5:395. doi:10.1186/1752-1947-5-395. 21854567 PMC3177918

[5] Messacar K, Asturias EJ, Hixon AM, Van Leer-Buter C, Niesters HGM, Tyler KL, Abzug MJ, Dominguez SR. 2018. Enterovirus D68 and acute flaccid myelitis-evaluating the evidence for causality. Lancet Infect Dis 18:e239–e247. doi:10.1016/S1473-3099(18)30094-X. 29482893 PMC6778404

[6] Greninger AL, Naccache SN, Messacar K, Clayton A, Yu G, Somasekar S, Federman S, Stryke D, Anderson C, Yagi S, Messenger S, Wadford D, Xia D, Watt JP, Van Haren K, Dominguez SR, Glaser C, Aldrovandi G, Chiu CY. 2015. A novel outbreak enterovirus D68 strain associated with acute flaccid myelitis cases in the USA (2012–14): a retrospective cohort study. Lancet Infect Dis 15:671–682. doi:10.1016/S1473-3099(15)70093-9. 25837569 PMC6027625

[7] Van Haren K, Ayscue P, Waubant E, Clayton A, Sheriff H, Yagi S, Glenn-Finer R, Padilla T, Strober JB, Aldrovandi G, Wadford DA, Chiu CY, Xia D, Harriman K, Watt JP, Glaser CA. 2015. Acute flaccid myelitis of unknown etiology in California, 2012–2015. JAMA 314:2663–2671. doi:10.1001/jama.2015.17275. 26720027

[8] Sejvar JJ, Lopez AS, Cortese MM, Leshem E, Pastula DM, Miller L, Glaser C, Kambhampati A, Shioda K, Aliabadi N, Fischer M, Gregoricus N, Lanciotti R, Nix WA, Sakthivel SK, Schmid DS, Seward JF, Tong S, Oberste MS, Pallansch M, Feikin D. 2016. Acute flaccid myelitis in the United States, August-December 2014: results of nationwide surveillance. Clin Infect Dis 63:737–745. doi:10.1093/cid/ciw372. 27318332 PMC5709818

[9] Solomon T, Ravi V. 2003. Acute flaccid paralysis caused by West Nile virus. Lancet Infect Dis 3:189–190. doi:10.1016/S1473-3099(03)00574-7. 12679260

[10] Chung CC, Lee SS, Chen YS, Tsai HC, Wann SR, Kao CH, Liu YC. 2007. Acute flaccid paralysis as an unusual presenting symptom of Japanese encephalitis: a case report and review of the literature. Infection 35:30–32. doi:10.1007/s15010-007-6038-7. 17297587

[11] Morens DM, Folkers GK, Fauci AS. 2019. Acute flaccid myelitis: something old and something new. mBio 10:e00521-19. doi:10.1128/mBio.00521-19. 30940708 PMC6445942

[12] Mateen FJ, Black RE. 2013. Expansion of acute flaccid paralysis surveillance: beyond poliomyelitis. Trop Med Int Health 18:1421–1422. doi:10.1111/tmi.12181. 24033476

[13] United Kingdom Acute Flaccid Paralysis (AFP) Task Force. 2019. An increase in reports of acute flaccid paralysis (AFP) in the United Kingdom, 1 January 2018–21 January 2019: early findings. Euro Surveill 24:1900093. doi:10.2807/1560-7917.ES.2019.24.6.1900093. PMC6373064 30755296

[14] Knoester M, Helfferich J, Poelman R, Van Leer-Buter C, Brouwer OF, Niesters HGM, 2016 EV-D68 AFM Working Group. 2019. Twenty-nine cases of enterovirus-D68-associated acute flaccid myelitis in Europe 2016: a case series and epidemiologic overview. Pediatr Infect Dis J 38:16–21. doi:10.1097/INF.0000000000002188. 30234793 PMC6296836

[15] Pérez G, Rosanova MT, Freire MC, Paz MI, Ruvinsky S, Rugilo C, Ruggieri V, Cisterna D, Martiren S, Lema C, Savransky A, González S, Martínez L, Viale D, Bologna R. 2017. Unusual increase of cases of myelitis in a pediatric hospital in Argentina. Arch Argent Pediatr 115:364–369. 28737865 10.5546/aap.2017.eng.364

[16] Labeaud AD, Bashir F, King CH. 2011. Measuring the burden of arboviral diseases: the spectrum of morbidity and mortality from four prevalent infections. Popul Health Metr 9:1. doi:10.1186/1478-7954-9-1. 21219615 PMC3024945

[17] World Health Organization. 2012. Weekly epidemiological record, 2012, p 161–168, vol 87. World Health Organization, Geneva, Switzerland.

[18] Laxmivandana R, Yergolkar P, Gopalkrishna V, Chitambar SD. 2013. Characterization of the non-polio enterovirus infections associated with acute flaccid paralysis in south-western India. PLoS One 8:e61650. doi:10.1371/journal.pone.0061650. 23630606 PMC3632520

[19] Liu W, Fu S, Ma X, Chen X, Wu D, Zhou L, Yin Q, Li F, He Y, Lei W, Li Y, Xu S, Wang H, Wang Z, Wang H, Yu H, Liang G. 2020. An outbreak of Japanese encephalitis caused by genotype Ib Japanese encephalitis virus in China, 2018: a laboratory and field investigation. PLoS Negl Trop Dis 14:e0008312. doi:10.1371/journal.pntd.0008312. 32453787 PMC7274457

[20] Maan HS, Dhole TN, Chowdhary R. 2019. Identification and characterization of nonpolio enterovirus associated with nonpolio-acute flaccid paralysis in polio endemic state of Uttar Pradesh, Northern India. PLoS One 14:e0208902. doi:10.1371/journal.pone.0208902. 30699113 PMC6353074

[21] Fall A, Ndiaye N, Messacar K, Kebe O, Jallow MM, Harouna H, Kiori DE, Sy S, Goudiaby D, Dia M, Niang MN, Ndiaye K, Dia N. 2020. Enterovirus D68 subclade B3 in children with acute flaccid paralysis in West Africa, 2016. Emerg Infect Dis 26:2227–2230. doi:10.3201/eid2609.200312. 32818390 PMC7454047

[22] Fang Y, Zhang Y, Zhou Z-B, Xia S, Shi W-Q, Xue J-B, Li Y-Y, Wu J-T. 2019. New strains of Japanese encephalitis virus circulating in Shanghai, China after a ten-year hiatus in local mosquito surveillance. Parasit Vectors 12:22. doi:10.1186/s13071-018-3267-9. 30626442 PMC6327439

[23] Narain JP, Dhariwal AC, MacIntyre CR. 2017. Acute encephalitis in India: an unfolding tragedy. Indian J Med Res 145:584–587. doi:10.4103/ijmr.IJMR_409_17. 28948947 PMC5644291

[24] Solomon T, Dung NM, Kneen R, Gainsborough M, Vaughn DW, Khanh VT. 2000. Japanese encephalitis. J Neurol Neurosurg Psychiatry 68:405–415. doi:10.1136/jnnp.68.4.405. 10727474 PMC1736874

[25] Gurav YK, Tandale BV, Jadi RS, Gunjikar RS, Tikute SS, Jamgaonkar AV, Khadse RK, Jalgaonkar SV, Arankalle VA, Mishra AC. 2010. Chandipura virus encephalitis outbreak among children in Nagpur division, Maharashtra, 2007. Indian J Med Res 132:395–399. 20966517

[26] Zimmerman HM. 1946. The pathology of Japanese B encephalitis. Am J Pathol 22:965–991. 20999309

[27] Misra UK, Kalita J, Jain SK, Mathur A. 1994. Radiological and neurophysiological changes in Japanese encephalitis. J Neurol Neurosurg Psychiatry 57:1484–1487. doi:10.1136/jnnp.57.12.1484. 7798977 PMC1073229

[28] Kumar S, Misra UK, Kalita J, Salwani V, Gupta RK, Gujral R. 1997. MRI in Japanese encephalitis. Neuroradiology 39:180–184. doi:10.1007/s002340050388. 9106289

[29] Jackson AC, Moench TR, Griffin DE, Johnson RT. 1987. The pathogenesis of spinal cord involvement in the encephalomyelitis of mice caused by neuroadapted Sindbis virus infection. Lab Invest 56:418–423. 3031369

[30] Samuel MA, Morrey JD, Diamond MS. 2007. Caspase 3-dependent cell death of neurons contributes to the pathogenesis of West Nile virus encephalitis. J Virol 81:2614–2623. doi:10.1128/JVI.02311-06. 17192305 PMC1866006

[31] Shrestha B, Gottlieb D, Diamond MS. 2003. Infection and injury of neurons by West Nile encephalitis virus. J Virol 77:13203–13213. doi:10.1128/jvi.77.24.13203-13213.2003. 14645577 PMC296085

[32] Wang YF, Chou CT, Lei HY, Liu CC, Wang SM, Yan JJ, Su IJ, Wang JR, Yeh TM, Chen SH, Yu CK. 2004. A mouse-adapted enterovirus 71 strain causes neurological disease in mice after oral infection. J Virol 78:7916–7924. doi:10.1128/JVI.78.15.7916-7924.2004. 15254164 PMC446098

[33] Goody RJ, Schittone SA, Tyler KL. 2008. Experimental reovirus-induced acute flaccid paralysis and spinal motor neuron cell death. J Neuropathol Exp Neurol 67:231–239. doi:10.1097/NEN.0b013e31816564f0. 18344914 PMC2365907

[34] Havert MB, Schofield B, Griffin DE, Irani DN. 2000. Activation of divergent neuronal cell death pathways in different target cell populations during neuroadapted Sindbis virus infection of mice. J Virol 74:5352–5356. doi:10.1128/jvi.74.11.5352-5356.2000. 10799613 PMC110891

[35] Ghosh S, Dutta K, Basu A. 2013. Chandipura virus induces neuronal death through Fas-mediated extrinsic apoptotic pathway. J Virol 87:12398–12406. doi:10.1128/JVI.01864-13. 24027318 PMC3807914

[36] Verma AK, Ghosh S, Pradhan S, Basu A. 2016. Microglial activation induces neuronal death in Chandipura virus infection. Sci Rep 6:22544. doi:10.1038/srep22544. 26931456 PMC4773833

[37] Mukherjee S, Akbar I, Kumari B, Vrati S, Basu A, Banerjee A. 2019. Japanese encephalitis virus-induced let-7a/b interacted with the NOTCH-TLR7 pathway in microglia and facilitated neuronal death via caspase activation. J Neurochem 149:518–534. doi:10.1111/jnc.14645. 30556910

[38] Swaroop S, Mahadevan A, Shankar SK, Adlakha YK, Basu A. 2018. HSP60 critically regulates endogenous IL-1β production in activated microglia by stimulating NLRP3 inflammasome pathway. J Neuroinflammation 15:177. doi:10.1186/s12974-018-1355-6. 29885667 PMC5994257

[39] Verma AK, Ghosh S, Basu A. 2018. Chandipura virus induced neuronal apoptosis via calcium signaling mediated oxidative stress. Front Microbiol 9:1489. doi:10.3389/fmicb.2018.01489. 30034380 PMC6043780

[40] Kaushik DK, Mukhopadhyay R, Kumawat KL, Gupta M, Basu A. 2012. Therapeutic targeting of Krüppel-like factor 4 abrogates microglial activation. J Neuroinflammation 9:57. doi:10.1186/1742-2094-9-57. 22429472 PMC3325890

[41] Misra UK, Kalita J. 1997. Movement disorders in Japanese encephalitis. J Neurol 244:299–303. doi:10.1007/s004150050090. 9178154

[42] Solomon T, Vaughn DW. 2002. Pathogenesis and clinical features of Japanese encephalitis and West Nile virus infections. Curr Top Microbiol Immunol 267:171–194. doi:10.1007/978-3-642-59403-8_9. 12082989

[43] Misra UK, Kalita J. 1997. Anterior horn cells are also involved in Japanese encephalitis. Acta Neurol Scand 96:114–117. doi:10.1111/j.1600-0404.1997.tb00250.x. 9272188

[44] Thach DC, Kimura T, Griffin DE. 2000. Differences between C57BL/6 and BALB/cBy mice in mortality and virus replication after intranasal infection with neuroadapted Sindbis virus. J Virol 74:6156–6161. doi:10.1128/jvi.74.13.6156-6161.2000. 10846099 PMC112114

[45] Pulit-Penaloza JA, Scherbik SV, Brinton MA. 2012. Type 1 IFN-independent activation of a subset of interferon stimulated genes in West Nile virus Eg101-infected mouse cells. Virology 425:82–94. doi:10.1016/j.virol.2012.01.006. 22305622 PMC3288888

[46] Ashley CL, Abendroth A, McSharry BP, Slobedman B. 2019. Interferon-independent upregulation of interferon-stimulated genes during human cytomegalovirus infection is dependent on IRF3 expression. Viruses 11:246. doi:10.3390/v11030246. PMC6466086 30871003

[47] Hiscott J, Paz S, Nakhaei P. 2011. CS09-7. Cross-talk between RIG-I dependent antiviral signalling and apoptosis. Cytokine 56:57. doi:10.1016/j.cyto.2011.07.359.

[48] Kaushik DK, Gupta M, Kumawat KL, Basu A. 2012. NLRP3 inflammasome: key mediator of neuroinflammation in murine Japanese encephalitis. PLoS One 7:e32270. doi:10.1371/journal.pone.0032270. 22393394 PMC3290554

[49] Mukherjee S, Sengupta N, Chaudhuri A, Akbar I, Singh N, Chakraborty S, Suryawanshi AR, Bhattacharyya A, Basu A. 2018. PLVAP and GKN3 are two critical host cell receptors which facilitate Japanese encephalitis virus entry into neurons. Sci Rep 8:11784. doi:10.1038/s41598-018-30054-z. 30082709 PMC6079088

[50] Hazra B, Chakraborty S, Bhaskar M, Mukherjee S, Mahadevan A, Basu A. 2019. miR-301a regulates inflammatory response to Japanese encephalitis virus infection via suppression of NKRF activity. J Immunol 203:2222–2238. doi:10.4049/jimmunol.1900003. 31527198

