# Supplementary figures and images for: Involvement of RIG-I Pathway in Neurotropic Virus-Induced Acute Flaccid Paralysis and Subsequent Spinal Motor Neuron Death
Source: mBio. 2021 Nov 16;12(6):e02712-21. doi: 10.1128/mBio.02712-21 (PMC8593677; doi:10.1128/mBio.02712-21)

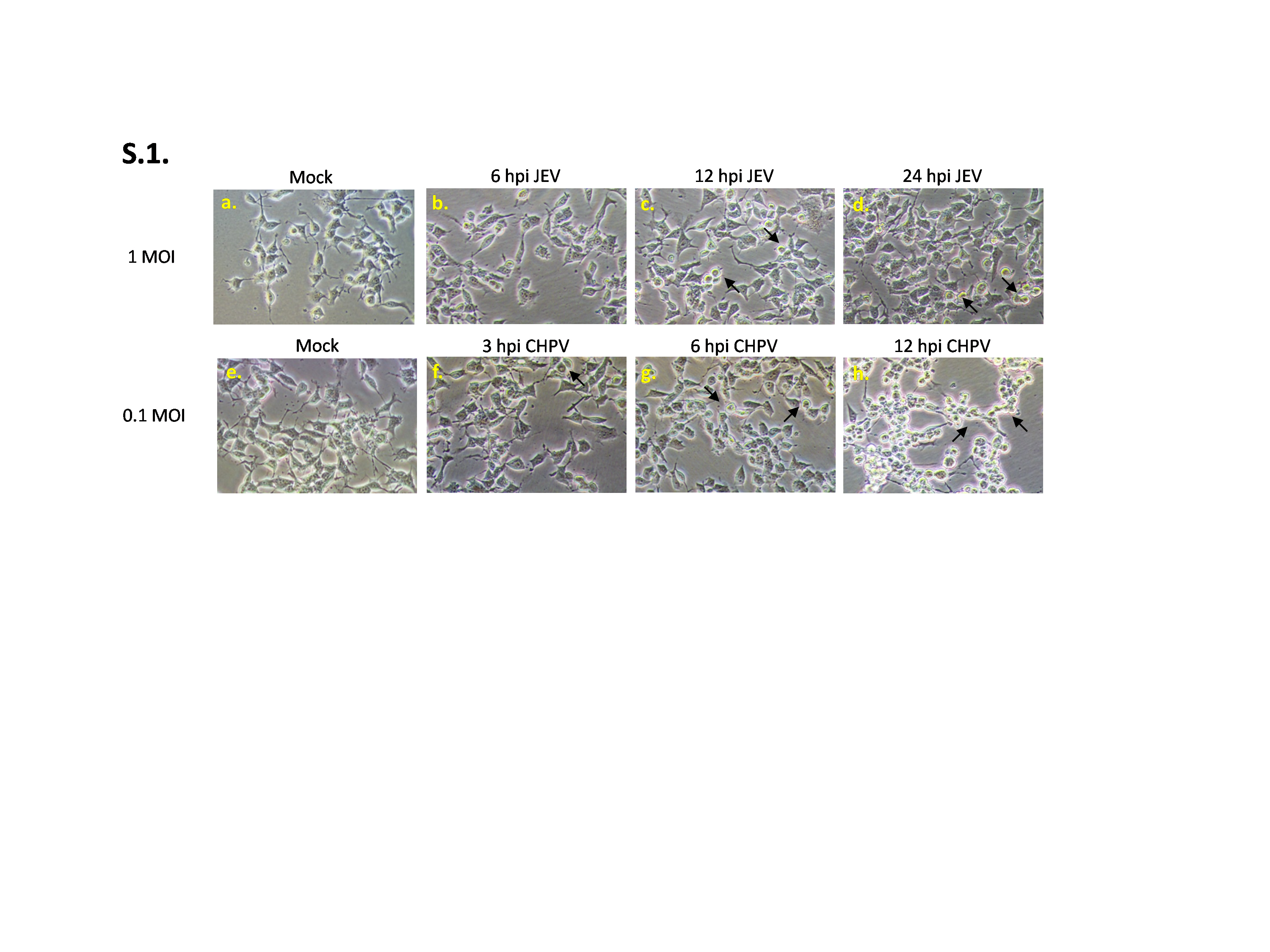

Supplement: FIG S1 [file mbio.02712-21-sf001.tif]

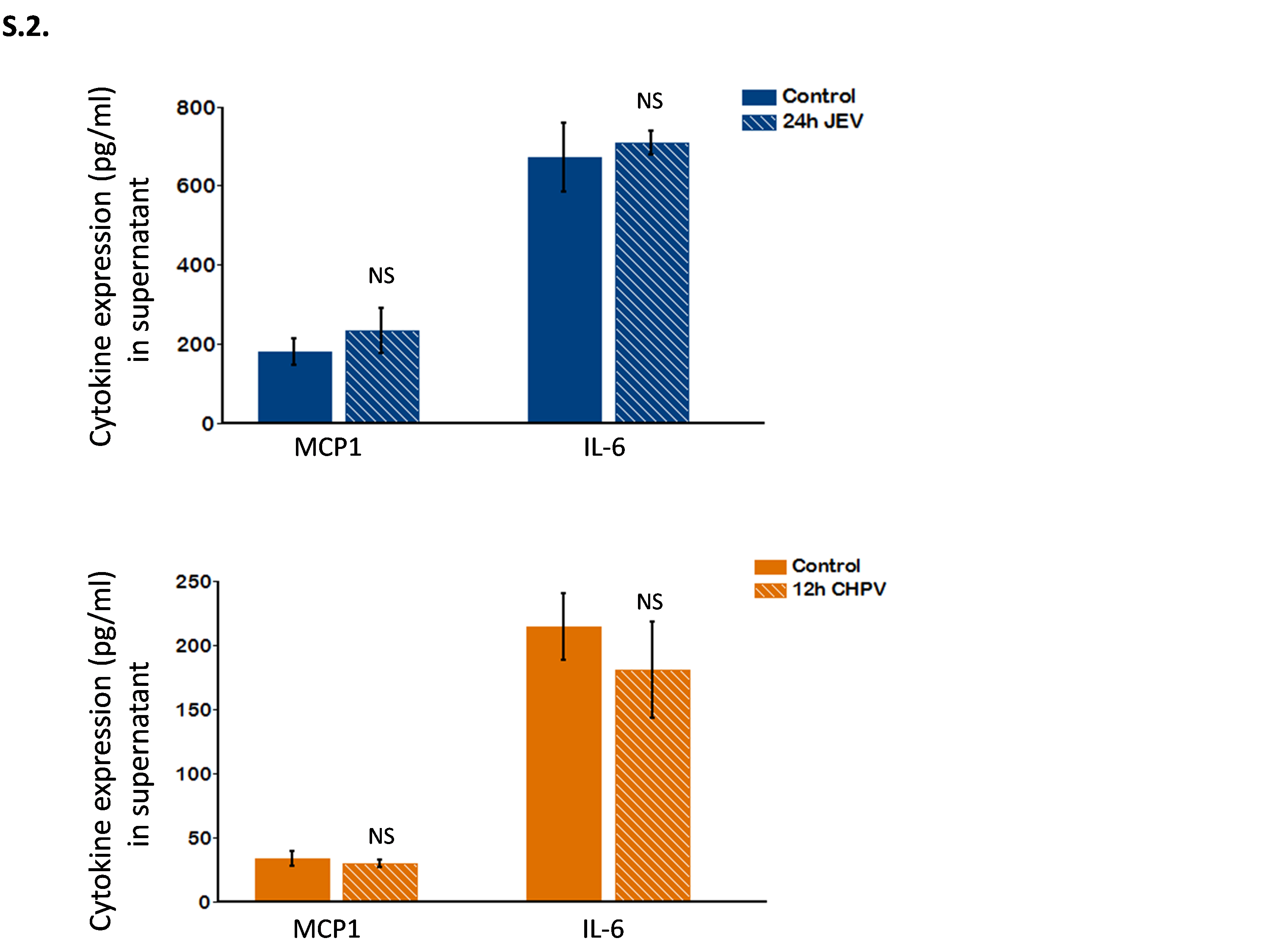

Supplement: FIG S2 [file mbio.02712-21-sf002.tif]

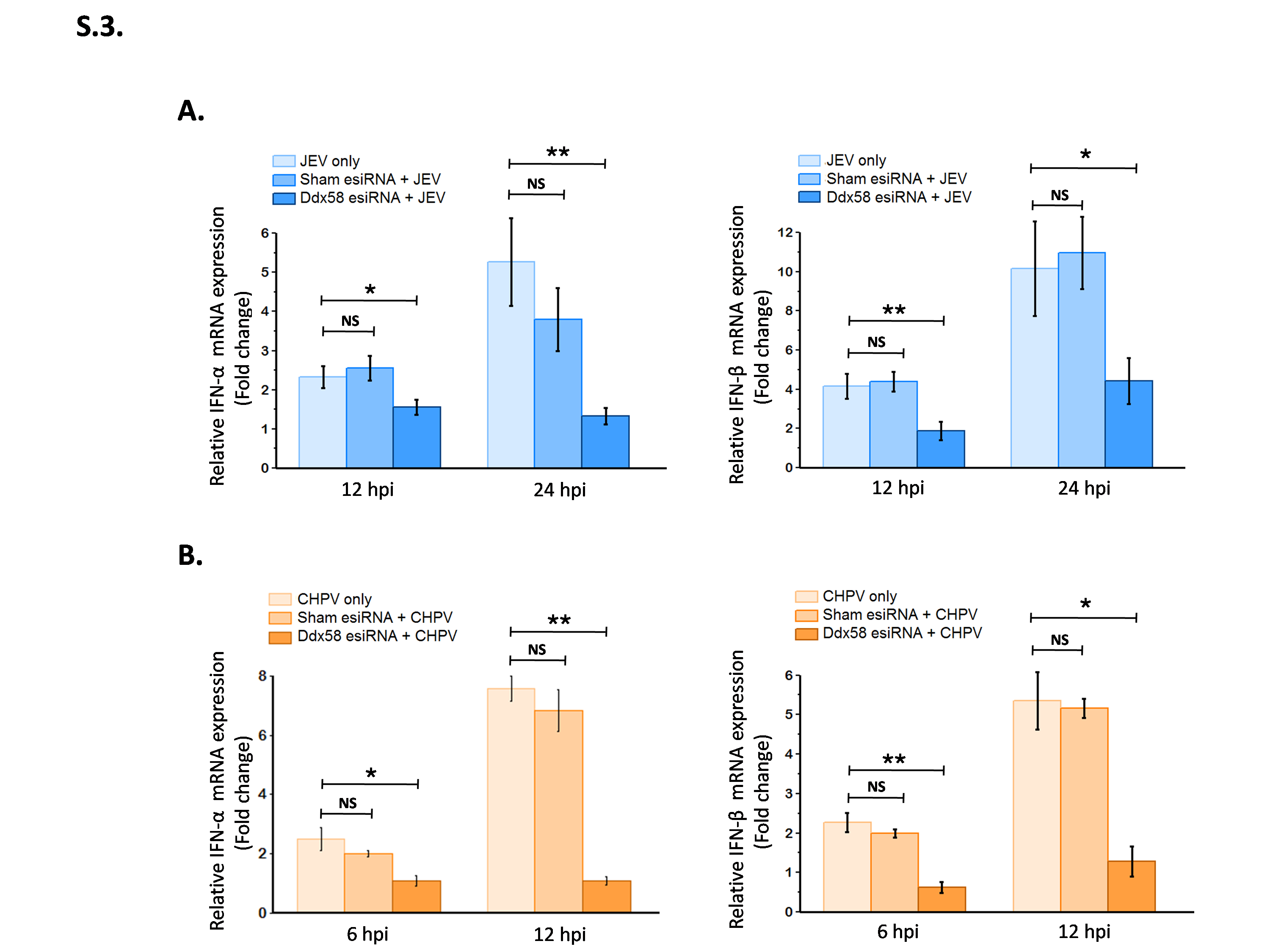

Supplement: FIG S3 [file mbio.02712-21-sf003.tif]

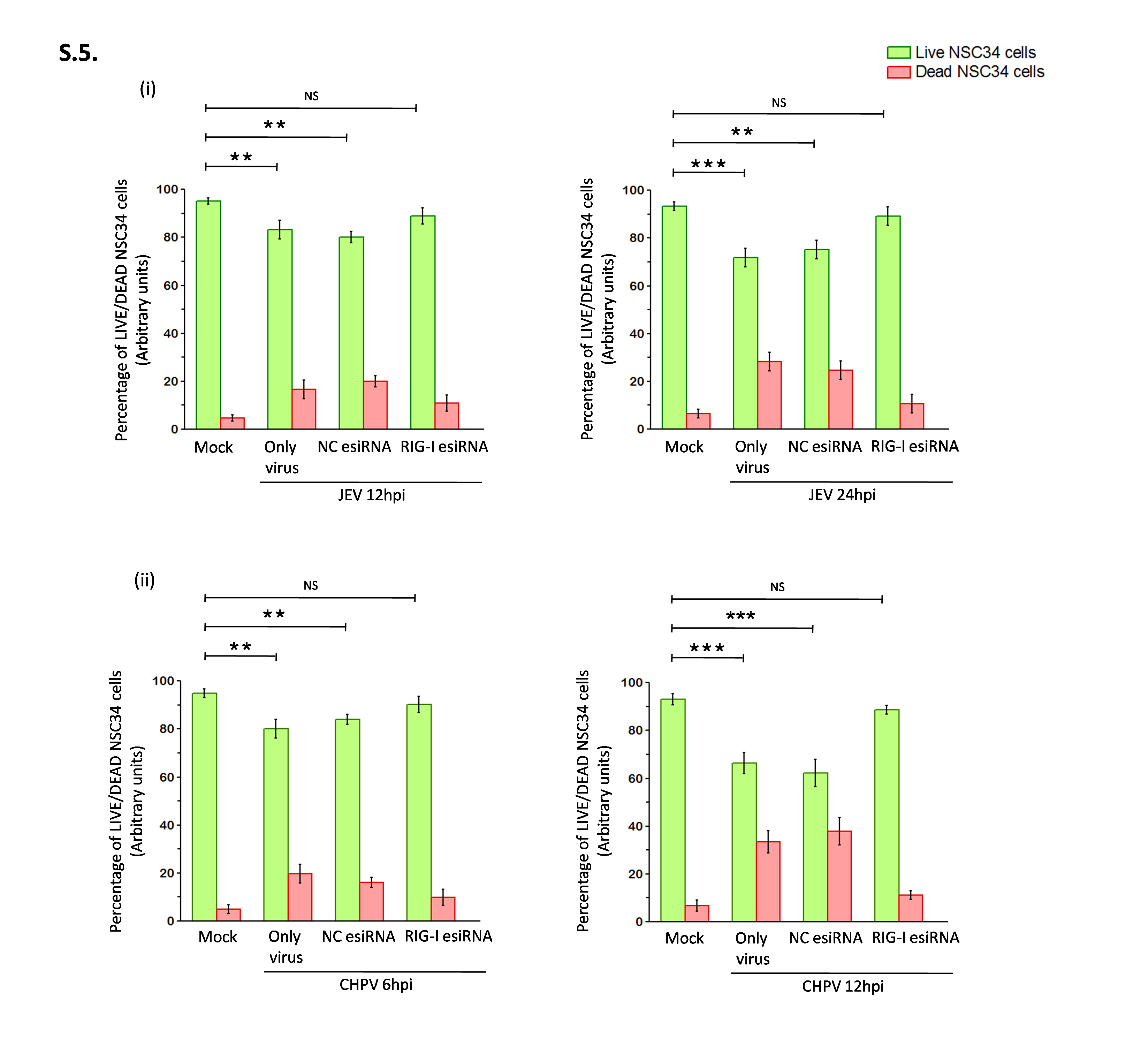

Supplement: FIG S5 [file mbio.02712-21-sf005.tif]

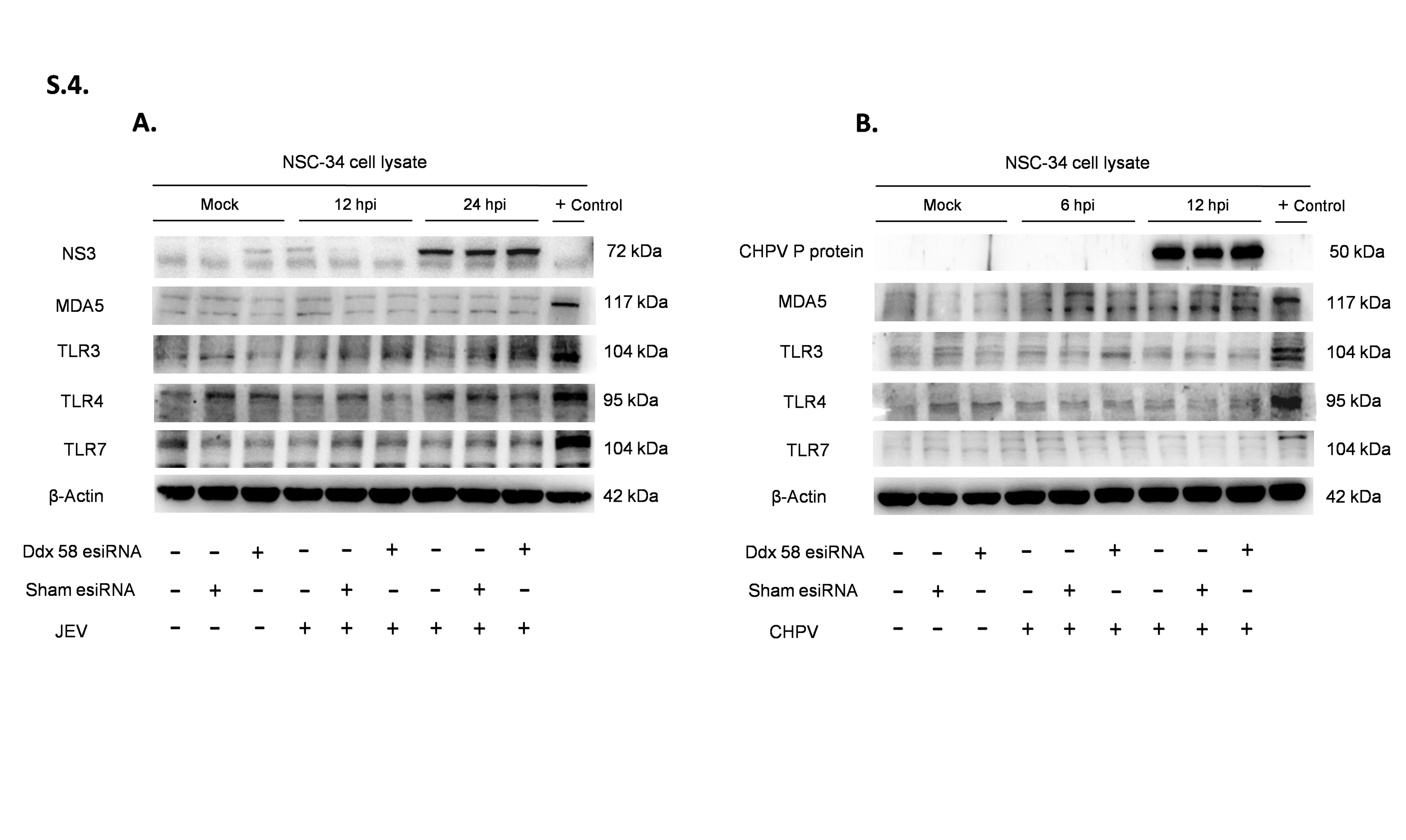

Supplement: FIG S4 [file mbio.02712-21-sf004.tif]
